# Targeting SRC enhances differentiation and promotes multifaceted cell death mechanisms in recurrent group 3 medulloblastoma

**DOI:** 10.1038/s41419-026-08751-9

**Published:** 2026-04-24

**Authors:** Helgi Kuzmychova, Ujala Chawla, Emma Martell, Akaljot Grewal, Charul Jain, Kayshana Ramnauth, Esha Kaul, Harshal Senthil, Chitra Venugopal, Christopher M. Anderson, Sheila K. Singh, Tanveer Sharif

**Affiliations:** 1 Department of Pathology, Rady Faculty of Health Sciences, University of Manitoba, Winnipeg, MB Canada; 2 Department of Pharmacology and Therapeutics, Rady Faculty of Health Sciences, University of Manitoba, Winnipeg, MB Canada; 3 Faculty of Science, University of Manitoba, Winnipeg, MB Canada; 4 College of Medicine, Rady Faculty of Health Sciences, University of Manitoba, Winnipeg, MB Canada; 5 Department of Surgery, McMaster University, Hamilton, ON Canada; 6 Centre for Discovery in Cancer Research, McMaster University, Hamilton, ON Canada; 7 PrairieNeuro Research Centre, Kleysen Institute for Advanced Medicine, Health Sciences Centre, Winnipeg, MB Canada; 8 Department of Biochemistry, McMaster University, Hamilton, ON Canada; 9 Department of Human Anatomy and Cell Science, Rady Faculty of Health Sciences, University of Manitoba, Winnipeg, MB Canada; 10 Paul Albrechtsen Research Institute, CancerCare Manitoba, Winnipeg, MB Canada

**Keywords:** Paediatric cancer, Cancer therapeutic resistance, Chemotherapy

## Abstract

Medulloblastoma (MB) is the most common childhood brain cancer, with Group 3 (G3) as the most aggressive subgroup, being prone to relapse and treatment resistance. A small subset of stem-like cells contributes to this recurrence, but the mechanisms behind their transformation are not fully understood. In this study, we employed therapeutically relevant in vitro and in vivo chemoradiotherapy (CRT) models of G3 MB and discovered a significant activation of SRC kinase following CRT treatment, while other kinases such as AKT and ERK were unaffected. Remarkably, SRC activation was exclusive to G3 MB cells and was absent in the less aggressive Sonic Hedgehog and Group 4 MB, as well as in normal brain cells. SRC activation in CRT-treated G3 MB cell and tumors corresponded with increased stemness, as evidenced by elevated levels of stemness factors SOX2, NOTCH1, OCT4, Nanog and phosphorylated STAT3, alongside a reduction in the differentiation marker βIII-tubulin/TUBB3. Conversely, *SRC* knockout or pharmacological inhibition promoted differentiation and reduced aggressiveness in CRT-resistant G3 MB cells, which could be rescued by re-expression of SRC in *SRC* knockout cells. Additionally, SRC inhibition significantly reduced the viability of CRT-treated G3 MB cells by inducing both apoptosis and necroptosis, while sparing the proliferation and stem-like properties of normal neural stem cells, indicating a promising toxicity profile. Importantly, in a therapeutically relevant orthotopic G3 MB model, administration of the re-purposed blood-brain-barrier permeable SRC inhibitor, Saracatinib, in conjunction with CRT, significantly reduced tumor burden and improved animal survival compared to CRT treatment alone without any neurotoxic side effects. Overall, our results underscore the pivotal role of SRC in enhancing stemness and aggressive behavior in CRT-resistant recurrent G3 MB. Targeting SRC not only promotes cell death through apoptosis and necroptosis but also encourages differentiation, positioning it as a promising therapeutic target for rapid clinical interventions.

## Introduction

Central nervous system tumors are the leading cause of cancer-related death in children, with medulloblastoma (MB) being the most common pediatric brain cancer [1, 2]. Proteogenomic profiling has classified MB into four distinct molecular subgroups, each differing in 5-year survival prognosis: Wingless (WNT) MB ( > 95%), Sonic Hedgehog (SHH) MB ( > 75%), Group 4 (G4) MB (60–75%), and, the most aggressive subgroup, Group 3 (G3) MB (40–58%) [3–6]. Although molecular classification presents a powerful prognostic tool, most MB patients older than 2 years receive the same multi-modal standard-of-care treatment involving surgery, radiation, and chemotherapy (cisplatin, vincristine, and/or cyclophosphamide) [7, 8]. Despite harsh treatment that often causes lifelong side effects in survivors, MB remains susceptible to relapse [9–12], with G3 exhibiting the highest rate of recurrence (>30%) and these treatment-resistant, metastatic recurrent tumors drive high mortality rates [11–13].

Standard therapies can eliminate most G3 MB cells, yet a small subset of poorly differentiated stem-like cells often survives and triggers relapse [13–15]. Notably, genomic analysis indicates that recurrent tumors retain their major genetic characteristics despite phenotypic changes [11, 12], suggesting that non-genomic changes in signaling pathways may play a crucial role in driving MB recurrence. A deeper understanding of signaling alterations in recurrent cells could illuminate potential targets for innovative treatments to mitigate G3 MB relapse.

Kinases are key signaling proteins that integrate signals from diverse stimuli and regulate cellular functions through protein phosphorylation [16–19]. Their frequent aberrant activation in tumors and the availability of numerous FDA-approved kinase inhibitors make them an appealing target for drug repurposing [20–23]. One notable kinase is SRC, a non-receptor protein kinase that regulates numerous cellular processes, including proliferation, adhesion, migration, differentiation, stemness, and both pro-survival and pro-apoptotic pathways, depending on the cellular environment [24–28]. Despite its potential as a therapeutic target, our understanding of the role SRC plays in G3 MB relapse and treatment resistance remains limited.

In this study, we identify SRC kinase activation as a crucial factor driving G3 MB relapse, as it sustains stemness and cell survival. Our findings demonstrate that genetic or pharmacological inhibition of SRC promotes cell differentiation and death through both apoptosis and necroptosis in recurrent and therapy-resistant G3 MB cells. Combining the repurposed SRC inhibitor, Saracatinib, and standard-of-care treatment significantly improves survival in a clinically relevant animal model, highlighting therapeutic potential. Moreover, inhibition of SRC spares the proliferation and stemness of normal brain cells, including astrocytes and neural stem cells (NSCs), providing a tumor-specific vulnerability.

## Materials and methods

### Ethics statement

The study protocol of mouse care and experiments was approved by the University of Manitoba’s Animal Care Committee (protocol #24-059). All animal studies complied with relevant ethical regulations for animal testing and research. Human NSCs were isolated from embryonic brain tissue specimens from a gestational age of 8–11 weeks. Embryonic material was donated voluntarily for research purposes by women who had a termination of pregnancy, as approved by the Hamilton Health Sciences/McMaster Health Sciences Research Ethics Board (REB; Project# 15530). Samples were collected with informed written consent from the mothers whose pregnancy was terminated in strict accordance with institutional and legal ethical guidelines. Patient-derived tumor samples and cell lines were also obtained with informed consent from the patient or the primary caregivers.

### Cell culture, patient demographics, and treatments

The sources and catalog numbers for all culture reagents and chemicals used in this study are listed in Supplementary Table 1.

Primary human pediatric MYC-amplified MB cell cultures were obtained from collaborators as kind gifts. Dr. Till Milde provided HD-MB03 [29], a treatment-naïve large cell/anaplastic G3 MB cell model isolated from a male patient $\le$ 3 years old during surgical intervention. SU_MB002 cells were provided by Dr. Yoon-Jae Cho and were derived from an autopsy specimen of the leptomeningeal compartment from a child with treatment-refractory, metastatic G3 MB after receiving only cyclophosphamide treatment [30]. MB3W1 anaplastic G3 MB cells were derived from the malignant cells found in the pleural effusions of a male patient ≤3-years old and kindly provided by Dr. Matthias Wölfl (Universitätsklinikum Würzburg) [31]. Dr. Sheila Singh kindly provided us with matched human pediatric primary D425 and recurrent D458 G3 MB cells [32]. The D283 G3 MB cell line was established from malignant ascites cells and a peritoneal metastasis from a male patient, G3/4 MB, between 6 and 10 years old, and was purchased from the American Type Culture Collection (ATCC, Rockville, MD, USA) [32, 33].

Dr. Sheila Singh kindly provided primary human pediatric SHH MB cells, BT992, previously isolated from a patient who later relapsed, with consent from the patient and families as approved by the Hamilton Health Sciences/McMaster Health Sciences Research Ethics Board [34]. DAOY cells, isolated from a 4-year old male patient with desmoplastic SHH MB [35], were purchased from the American Type Culture Collection (ATCC, Rockville, MD, USA). All cells were maintained in a humidified incubator at 37 °C with 5% CO_2_ for a maximum of 20–25 passages. HD-MB03, SU_MB002, and MB3W1 cells were cultured as tumorspheres in serum-free brain-tumor initiating cell (BTIC) enrichment medium composed of knockout DMEM/F12 media (Life Technologies, Burlington, ON, Canada) with 1X GlutaMAX (Life Technologies), 20 ng/mL of EGF (STEMCELL Technologies, Vancouver, BC, Canada), 10 ng/mL of bFGF (STEMCELL Technologies), 1X NeuroCult SM1 Supplement (STEMCELL Technologies), 1X N2 Supplement (STEMCELL Technologies), 2 µg/mL Heparin (STEMCELL Technologies), and 1X antibiotic-antimycotic (Life Technologies). D425 cells were expanded in MEM media (Life Technologies) supplemented with 1000 mg/L D-glucose (Life Technologies), 1X antibiotic-antimycotic, 20% fetal bovine serum (FBS; Life Technologies), and 1 mM sodium pyruvate (Life Technologies). D458 cells were expanded in DMEM, high glucose media (Life Technologies) supplemented with 1X GlutaMax, 1X antibiotic-antimycotic, 10% FBS, and 1 mM sodium pyruvate. D283 cells were expanded in MEM media (Life Technologies) with 1X antibiotic-antimycotic, 10% FBS, and 1 mM sodium pyruvate. BT992 cells were expanded in BTIC enrichment medium supplemented with 10% FBS. DAOY cells were expanded in DMEM, high glucose media supplemented with 1X GlutaMAX, 1 mM sodium pyruvate, 1X non-essential amino acids (NEAA; Life Technologies), and 1X antibiotic-antimycotic. D425, D458, D283, BT992 and DAOY cells were cultured in serum-free BTIC enrichment medium for at least 48 h prior to experiments.

Human NSCs were isolated from embryonic brain tissue specimens at an age of 8–11 weeks. Samples were collected with informed written consent from patients in strict accordance with institutional and legal ethical guidelines. These cells were provided to the Sharif lab by Dr. Sheila Singh via a material transfer agreement (MTA; number: MTO20-120). Samples were dissociated in artificial cerebrospinal fluid containing 0.2 Wunisch U/mL Liberase (Roche), and incubated at 37 °C in a shaker for 15 min. The dissociated tissue was then filtered through a 70μm cell strainer and collected by centrifugation at 1500xrpm for 3 min as previously described [36] and cultured in serum-free DMEM/F12 media (Life Technologies) with 20 ng/mL of EGF, 10 ng/mL of bFGF, 1X NeuroCult SM1 Supplement, 1X N2 Supplement, 2 µg/mL Heparin, and 1X antibiotic antimycotic [37]. Human astrocytes were purchased from ScienCell Research Laboratories (Carlsbad, CA, USA) and were maintained in complete Astrocyte Medium (ScienCell Research Laboratories).

No commonly misidentified cell lines were used in this study, and the cell lines were authenticated by STR profiling (ATCC). Information on cell lines is also available in Supplementary Table 1. All cell lines tested negative for Mycoplasma contamination using the MycoAlert® Mycoplasma Detection Kit (Lonza, Basel, Switzerland).

### CRISPR-Cas9 gene editing

Tetracycline-inducible (Tet-ON) Cas9 lentivirus particles were purchased from Dharmacon (Horizon Discovery, Cat No. VCAS11227). 5 × 10^4^ G3 MB cells were seeded in a 24-well plate and immediately transduced with lentivirus particles at an MOI of 0.3 and 5ug/mL of sequabrene (Millipore Sigma, Burlington MA, USA). The plate was centrifuged at 800 × *g* for 1 h at 32 °C to improve transduction efficiency. Following centrifugation, cells were incubated at 37 °C with 5% CO_2_. 24 h following transduction, media with virus was removed from cells, and fresh media containing 8 µg/mL of blasticidin (Invitrogen) selection marker was added. Two weeks after selection, cells were transduced with two different lentivirus clones containing pre-designed guide RNA (gRNA) purchased from Dharmacon against *SRC* (clone #1: Cat. No VSGHSM_38240064, and clone #2: VSGHSM_38240069). The same transduction protocol was followed, and cells expressing gRNA were selected in 2 µg/mL of puromycin (Millipore Sigma). Cells were continuously maintained in media containing specified selection markers. To induce Cas9 expression and CRISPR gene-editing, 0.5 µM doxycycline hyclate (DOX; Millipore Sigma) was administered to cells daily for three consecutive days.

### In vitro treatments

For clinically relevant in vitro CRT treatment, cells were seeded (day 0) and exposed to 2 Gray units (Gy) of X-ray irradiation (Rad Source 2000) 24 h after plating (day 1). After a two-day rest period, on day 4, cells were treated with chemotherapeutic agents: cisplatin (200 nM; MedChemExpress, Monmouth Junction, NJ, USA) and vincristine (0.5 nM; MedChemExpress). On day 8, live cells were separated by magnetic bead sorting as described below.

Therapy-naïve cells received equivalent volumes of solvents without drugs as a vehicle control. To confirm the acquisition of treatment resistance, cells were challenged with either re-irradiation (2 Gy), high-dose cisplatin (500 nM), or vincristine (1 nM) chemotherapy treatment. For SRC inhibition, MB cells were treated with 10 µM Saracatinib (MedChemExpress), 5 µM Bosutinib (Selleckchem, Houston, TX, USA), or 5 µM SU6656 (Tocris Bioscience, Bristol, UK), and samples were collected after 24 h. To inhibit cell death pathways, cells were treated with 10 µM of N-benzyloxycarbonyl-Val-Ala-Asp(OMe)-fluoromethylketone (Z-VAD-FMK; Selleckchem) and 10 µM Necrostatin-1 (Nec-1; Selleckchem), and samples were collected after 24 h. Sources and catalog numbers for all chemicals used in this study are listed in Supplementary Table 1.

### Live cell separation

Living cells were isolated using the Dead Cell Removal Kit (Miltenyi Biotec, Bergisch Gladbach, North Rhine-Westphalia, Germany) according to the manufacturer’s protocol. Cells were pelleted by centrifugation at 600 x *g* for 5 min, and pellets were washed in 1 mL of 1X Binding Buffer. Cells were counted by trypan blue (Millipore Sigma) exclusion and resuspended in 100 µL of Dead Cell Removal MicroBeads per 10⁷ total cells and incubated for 15 min at room temperature. Following incubation, samples were loaded onto the LS MACS® Column in a MACS magnetic separator (Miltenyi Biotec), and the flow-through, containing the enriched live cell population, was collected. Cell viability was assessed using trypan blue exclusion, confirming that most of the cells were alive following separation. Sources and catalog numbers for all kits and reagents used in this study are listed in Supplementary Table 1.

### Transfection

For protein overexpression (OE), G3 MB cells were plated in 6-well ultra-low attachment plates at a density of 5 × 10^5^ cells per well. To restore SRC expression in SRC knockout cells, cells were treated with 0.5 µM doxycycline for 24 h to induce knock-out before receiving 250 µL of plasmid DNA with lipofectamine 3000 and P3000 complex (Invitrogen, Cat No. L3000015) prepared in Opti-MEM media according to the manufacturer’s instructions. Cells received 0.5 µg/mL of *SRC* (SRC (untagged)-Human v-src sarcoma (Schmidt-Ruppin A-2) viral oncogene homolog (avian) (SRC), transcript variant 1, OriGene, Cat No. SC125208) or empty vector EV (pCMV6-Entry Mammalian Expression Vector, OriGene, Cat No. PS100001). Following transfection, cells were treated with 0.5 µM doxycycline for 48 more h before sample collection.

### Human phospho-kinase array profiling

Phospho-kinase array profiling was performed using the Proteome Profiler Human Phospho-Kinase Array Kit (R&D Systems, Minneapolis, MN, USA) according to the manufacturer’s specifications and guidelines. Briefly, cells were lysed using the provided buffer, supplemented with protease and phosphatase inhibitors. Protein concentration was quantified using the colorimetric Micro BCA assay kit (Life Technologies), and 300 µg of total protein was incubated with the pre-blocked array membranes overnight at 4 °C on a rocking platform. The following day, membranes were washed (3 × 10 mins) and incubated with the provided antibody detection cocktail for 1 h at room temperature. Membranes were washed (3 × 10 mins) and incubated with the provided streptavidin-HRP reagent for 30 min at room temperature. Membranes were washed (3 × 10 mins) and covered with the chemiluminescent detection reagent mix provided in the kit. Membranes were imaged using the iBright FL1500 Imaging System with several exposure times to detect the optimal chemiluminescent signal. Kinase spot intensities were quantified using ImageJ software (National Institutes of Health) and normalized to internal reference spots on the membrane. Sources and catalog numbers for all chemicals used in this study are listed in Supplementary Table 1.

### Protein extraction and western immunoblotting

Suspension cells were collected and centrifuged at 500 x *g* for 5 min at 4 °C. Adherent cells were scraped in cold 1X PBS at pH 7.4 and centrifuged at 500 × *g* for 5 min at 4 °C. Pellets were resuspended in RIPA lysis buffer (25 mM Tris pH 7.6, 150 mM NaCl, 1% NP-40, 1% sodium deoxycholate, 1% SDS) containing 1X protease and phosphatase inhibitor cocktail (PIC; Life Technologies). Whole cell lysates were incubated on ice for 45 min and then sonicated for 1 min. The samples were centrifuged at 20,000 x *g* for 15 min at 4 °C and the supernatants containing the proteins were collected. Protein concentrations were determined using the colorimetric Micro BCA assay kit (Life Technologies) according to the manufacturer’s instructions. Equal amounts of protein were diluted 1:1 in 2X Laemmli sample buffer (Bio-Rad, Hercules, CA, USA) containing 5% β-mercaptoethanol (Millipore Sigma), boiled for 5 min at 95 °C, and then resolved by SDS-PAGE. Protein was transferred onto 0.45 µm nitrocellulose membranes (Bio-Rad). To detect total protein loaded for normalization, membranes were labeled with No-Stain™ Protein Labeling Reagent (Invitrogen) according to the manufacturer’s instructions. Membranes were then blocked in 5% non-fat milk in PBST (PBS + 0.05% Tween 20; Life Technologies) for 45 min, then washed in PBST (3 × 5 min) and incubated in the appropriate primary antibody overnight at 4 °C with shaking. The primary antibodies were prepared at a 1:1000 dilution in 1% BSA in PBST. The following day, membranes were washed in PBS (3×5 min) before incubating in appropriate horseradish peroxidase (HRP)-conjugated secondary antibodies. Secondary antibodies were prepared at a 1:10 000 dilution in 5% non-fat milk in PBS and added to the membranes for 1.5 h at room temperature. Details of specific primary and secondary antibodies used in this study and their dilutions can be found in Supplementary Table 2. Following secondary antibody incubation, membranes were washed (3 × 5 min), and proteins were detected using Clarity ECL Western substrate (Bio-Rad) and imaged using the iBright™ FL1500 Imaging System chemiluminescent setting. Sources and catalog numbers for all kits and reagents used in this study are listed in Supplementary Table 1. Semi-quantitative analysis of the protein densitometry signal was performed using ImageJ software, according to previously published guidelines [36, 38, 39]. Whole lane total protein normalization was performed using the No-Stain stain from the same gel as the respective protein of interest was probed on. Fold-change values were calculated relative to the respective control group, and the mean fold-change values were plotted from biological replicates spanning multiple independent experiments. Original uncropped Western blot images are provided in the supplementary material.

### Cell count

To monitor cell growth and viability, cells were stained with trypan blue and counted to determine the viability of cells in suspension. Trypan blue is a dye that stains dead cells due to their breached membrane integrity, allowing to discriminate non-viable cells. 2 × 10^4^ cells were seeded in 12-well plates. 24 h after seeding, cells were treated with the designated drug or vehicle control. At indicated time points, cells were collected and re-suspended in 1X PBS and mixed 1:1 with trypan blue and viable cells were counted using a hemocytometer. Sources and catalog numbers for all reagents used in this study are listed in Supplementary Table 1.

### Cell viability

Cell viability was assessed using the PrestoBlue HS Cell Viability Reagent (Life Technologies) according to the manufacturer’s protocol. Cells were seeded in 96-well plates at a density of 1000 cells per well in 100 µL of BTIC enrichment medium. 24 h after seeding, cells were treated with drugs or vehicle control at the indicated concentrations. 48 h following treatment, 10 µL of PrestoBlue reagent was added directly to each well and incubated at 37 °C for 15 min in a humidified incubator with 5% CO₂. Fluorescence was then measured using a microplate reader (excitation: 560 nm, emission: 590 nm). Background signal from media-only wells was subtracted, and data were normalized to vehicle-treated control wells to calculate the fold-change in cell viability. Sources and catalog numbers for all reagents used in this study are listed in Supplementary Table 1.

### Tumorsphere formation

Cells were dissociated into single cell suspensions through gentle trituration by pipetting and seeded at a low density of 5 × 10^3^ cells/well on ultralow-attachment 12-well plates in serum-free media. 24 h after seeding, cells were treated with drugs or vehicle control at the indicated concentrations. Images of tumorspheres were taken 5 days after initial seeding using a light microscope from multiple fields of view. For secondary sphere formation, primary tumor spheres were dissociated, counted, re-seeded at a density of 5 × 10^3^ cells/well on ultralow attachment 12-well plates in serum-free media, and imaged again after 5 days. The number of spheres with a diameter equal to or larger than 50 µm was quantified using ImageJ software. Sources and catalog numbers for all supplies and reagents used in this study are listed in Supplementary Table 1.

### Migration assay

Cell migration was assessed using a Transwell assay. eGFP-expressing G3 MB cells were seeded at a density of 2 × 10^4^ cells per well in the upper chamber of a Corning Transwell insert (Thermo Fisher Scientific) that contained MB media. The lower chamber was filled with 600 µL of MB media supplemented with 10% FBS, serving as a chemoattractant. Cells were treated according to the experimental conditions and incubated for 72 h. After incubation, the migrated GFP⁺ cells on the lower surface of the membrane were visualized using an EVOS® FL Cell Imaging System across multiple randomly selected fields. The number of migrated cells was quantified using ImageJ software (National Institutes of Health).

### Animal studies

All experiments involving animals were approved by the University of Manitoba’s Animal Care Committee (Protocol #24-059). Non-obese diabetic (NOD) severe combined immunodeficient (SCID) IL2R gamma null (NSG) mice (NOD.Cg-*Prkdc*^*scid*^ *Il2rg*^*tm1Wjl*^/SzJ) were acquired from the CancerCare Manitoba in-house breeding colony, courtesy of Dr. Jody Haigh. Animals were housed in IVC caging and held according to the Guidelines of the Canadian Council on Animal Care and the Animal Care and Use Policy of the University of Manitoba.

Irradiated feed was used, and caging and bedding were sterilized by steam autoclave. Animals had continuous access to food and water. Room ambient temperature was 21–23 °C with a relative humidity target of 50%, but within a range of 30–60%. Light cycle was 12 h on/12 h off, beginning with lights on at 6:00 a.m.

For modeling the progression of recurrent G3 MB, a total of 20, 7–9 week-old female NSG mice were anesthetized with isoflurane (5% induction, 2.5% maintenance) and 1 × 10⁵ HD-MB03 or SU_MB002 cells suspended in 5 µL of PBS were injected into the cerebellum using a stereotactic frame in a nonrandomized, nonblinded fashion with *n* = 10 mice per G3 MB cell line. This is a highly aggressive brain tumor model, and based on our experience, we know that animals develop visible tumors by 5 days post-engraftment [38]. On day 7 post-engraftment, animals (*n* = 5 per treatment group) were randomly assigned to receive placebo treatment with no drugs (Therapy-naïve controls) or CRT treatment. No blinding was done for animal studies. Mice designated to receive CRT treatment were subjected to 2 Gy of craniospinal X-ray irradiation on day 7 post-engraftment using the Rad Source 2000, with a cerrobend shield limiting exposure to the cranium and upper spine. One week following radiation (day 14 post-engraftment), cisplatin (2.5 mg/kg) and vincristine (0.4 mg/kg) treatment were administered to mice by intraperitoneal (I.P.) injection. Placebo control mice received volume-matched vehicle saline I.P. injections. The University of Manitoba’s Animal Care Committee has no maximal tumor size/burden restrictions for orthotopic brain tumor studies. Animals were sacrificed at humane endpoints as defined by 20% reduction from peak body weight or significant clinical deterioration (i.e., evidence of pain, neurological symptoms, paralysis, etc.) as decided in consultation with the veterinarian, regardless of tumor size/burden. Once the endpoint was reached, animals were perfused with formalin and brains were harvested and preserved in formalin for at least 5-7 days prior to histopathology. Formalin-fixed brains were sliced, paraffin-embedded, and cut to prepare tissue slides for immunostaining.

For survival studies, a total of 15, 7–9 week old female NSG mice were anesthetized with isoflurane (5% induction, 2.5% maintenance) and 1 × 10⁵ HD-MB03 cells suspended in 5 µL of PBS were injected into the cerebellum using a stereotactic frame in a nonrandomized, nonblinded fashion. On day 7 post-engraftment, animals were randomized into three treatment groups (*n* = 5 mice per group): 1. Placebo control, 2. CRT treatment, and 3. CRT + Saracatinib combination treatment. No blinding was done for animal studies. Mice designated to receive CRT treatment were treated as described above. Animals in the combination treatment group were treated with 40 mg/kg of Saracatinib, prepared in a 0.5% methylcellulose suspension, by oral gavage every other day (QOD), beginning one day after irradiation (day 8) until the end of the study. Placebo control mice received volume-matched vehicle saline I.P. injections and 0.5% methylcellulose oral gavage treatments containing no drugs. For survival outcomes, animals were weighed and monitored daily for general well-being and euthanized at humane endpoints as described above. One mouse assigned to the CRT-treatment group died from complications unrelated to the experimental protocol and was removed from subsequent analysis. Once the endpoint, as defined above, was reached, animals were perfused with formalin, and brains were harvested and preserved in formalin for at least 5–7 days prior to histopathology. Formalin-fixed brains were sliced, paraffin-embedded, and cut to prepare tissue slides for immunostaining.

T2 MRI imaging was performed using an MR Solutions cryogen-free FlexiScan 7 T system (MR Solutions, Guildford, Surrey, UK) to monitor tumor size. For tumor volume calculations from MRI images, the ImageJ freehand tool was used to determine the volume from 18 serial sections (300 μm thickness) from each sample, and the sum of all of the slices was then used to calculate an overall tumor volume.

### Tissue processing and immunostaining

Following deparaffinization of formalin-fixed and paraffin-embedded (FFPE) tissue samples, antigen retrieval was performed in citrate buffer at 95–100 °C for 20 min. Slides were washed with 1X PBS and blocked with 10% sheep serum, then incubated with primary antibodies overnight at 4 °C. Slides were washed with 1X PBS + 0.1% Tween 20 and incubated with fluorophore-conjugated secondary antibodies for 2 h at room temperature. Details of specific primary and secondary antibodies used in this study and their dilutions can be found in Supplementary Table 2. Finally, coverslips were mounted with Prolong™ Diamond Antifade Mountant with DAPI (Life Technologies). Images were captured using a Zeiss AxioImager Z2 in the Quantitative Imaging, Phenotyping and Sorting (QuIPS) Platform located in the Paul Albrechtsen Research Institute, CancerCare Manitoba and quantification of fluorescent intensity was performed using ImageJ. For the total fluorescent stain intensity evaluation, full scans of the tumor tissue were used. Tumor tissues were identified as regions of interest under DAPI DNA stain guidance, and integrated signal density in the appropriate antibody channel from all tumor regions per slide was summed up and normalized to the total area of all tumor regions. Sources and catalog numbers for reagents used in this study are listed in Supplementary Table 1.

### Bielschowsky silver staining

Neuronal fibers were visualized using the Bielschowsky Silver Stain Kit (Abcam, Cambridge, UK, Cat. No. ab245880) according to the manufacturer’s protocol. After deparaffinization, the slides were rehydrated in distilled water. A staining jar containing 25 mL of 20% Silver Nitrate Solution was pre-warmed in a water bath for 10 min, and slides were incubated for 15 min at 40 °C. Tissue slides were rinsed in 4 changes of distilled water for 5 min each, then incubated in Ammoniacal Silver Solution for 10 min at 40 °C. Afterward, slides were placed in the Developer Solution with gentle agitation until a yellow-brown coloration appeared (5–20 s), then transferred to Ammonia Water for 30 s. Slides were fixed in 5% Sodium Thiosulfate Solution for 2 min, rinsed, dehydrated in ethanol, and mounted using coverslips and Permount (Fisher Scientific). Images were captured using a Zeiss Axio Imager.

### Bioinformatic analysis

Publicly available data set with gene expression in paired primary and recurrent MB tumors produced by Okonechnikov et al. [40] was accessed from the R2 platform (http://r2.amc.nl) under the name “Tumor Medulloblastoma—Korshunov—86—rpkm—mbffpe”. Normalized gene expression data were downloaded from the R2 platform and processed in R and R Studio using packages markeR, ggplot2, and ggpubr. GAUTSCHI_SRC_SIGNALING GSEA molecular signature was used to assess SRC activity. We quantified the effect size of the change in the SRC activity signature using Cohen’s *d*, which measures the standardized difference between two group means. Conventionally, values ~ 0.2 indicate a small effect, ~0.5 medium, and ~0.8 large. Positive values indicate that the first group has a higher mean, while negative values indicate that the second group has a higher mean.

### Statistical analysis

Statistical analysis was performed in GraphPad Prism 10. Statistical parameters, including the exact value of n and the statistical significance, are reported in the figures and figure legends. No power calculations were utilized to determine the required sample size for in vitro and in vivo experiments. For in vivo studies, experiments included an equal number of mice in each treatment group with *n* = 5 animals per experimental condition. One animal was excluded from the analysis as it died from complications unrelated to the study protocols. For in vitro studies, all experiments were performed on a minimum of 3 independent biological replicates. No sample size calculation was made for analysis of data accessed from public repositories, as all available cases in the cited repositories were included.

Data were tested for normality using the Shapiro-Wilk test and confirmed to be normally distributed. To assess significant differences between single measurements of two groups of normally distributed data, the unpaired two-tailed Student’s *t* test was used. To assess significant differences between more than two groups of normally distributed data, we performed one-way or two-way analysis of variance (ANOVA), followed by either a Fishers Least Significant Difference (LSD) test or when all pairs of datasets were compared, Tukey’s multiple comparisons test was performed and when every mean was only compared to the control mean, Dunnett’s multiple comparisons test was performed. The data are presented as mean + standard error of the mean (SEM) unless otherwise specified. Differences were considered statistically significant at *P* < 0.05.

## Results

### SRC phosphorylation is increased in therapy-resistant G3 MB cells

**Fig. 1:**
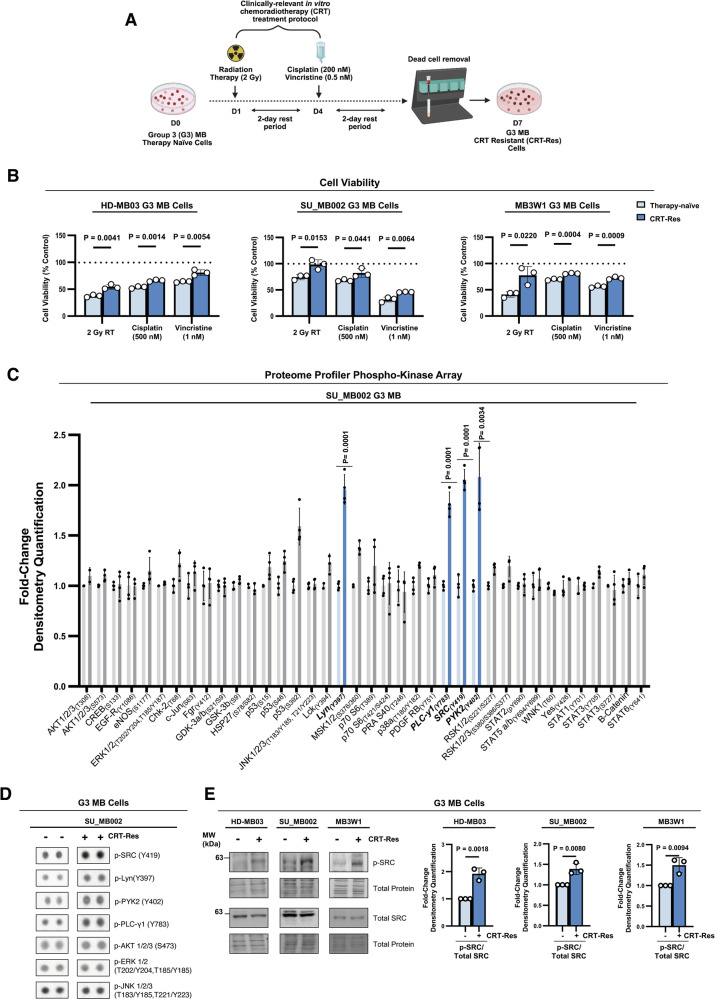
SRC phosphorylation is increased in therapy-resistant G3 MB cells. **A** Schematic diagram depicting the in vitro CRT treatment regimen used in this study, generated using Biorender. **B** Viability of therapy naïve and CRT-Res HD-MB03, SU_MB002, and MB3W1 G3 MB cells following treatment with 2 Gy RT, Cisplatin (500 nM), or Vincristine (1 nM) measured using PrestoBlue. Data represent mean + s.e.m; from *n* = 3 experimental replicates. Unpaired two-tailed t-tests. **C** Quantification of Proteome Profiler Phospho-Kinase array results from *n* = 4 experimental replicates, densitometry quantification measurement normalized to the internal membrane reference spots, presented as mean ± s.e.m; unpaired two-tailed t-tests. **D** Representative dot blots of p-SRC (Y419), p-Lyn (Y397), p-PYK2 (Y402), p-LC-γ1(Y783), p-AKT1/2/3 (S473), p-ERK1/2 (T202/Y204, T185/Y185), and p-JNK1/2/3 (T183/Y185, T221/Y223) spots from the phospho-kinase array membranes comparing therapy-naïve and CRT-Res SU_MB002 G3 MB cells. **E** Immunoblot of p-SRC (Y419) and total SRC in therapy naïve and CRT-Res HD-MB03, SU_MB002, and MB3W1 G3 MB. Graphs represent densitometry quantification measurement normalized to the total protein stain of p-SRC/total SRC from *n* = 3 experimental replicates presented as mean + s.e.m; unpaired two-tailed t-tests.

To investigate non-genetic signaling adaptations mediating therapy resistance in G3 MB, we utilized a clinically relevant CRT treatment protocol adapted for cell culture that closely mimics standard-of-care therapy. Three distinct G3 MB cell lines (SU_MB002, HD-MB03, and MB3W1) recently isolated from patient tumors [29–31], were exposed to 2 Gy of radiation therapy (RT), followed by concurrent treatment with cisplatin (200 nM) and vincristine (0.5 nM) chemotherapies [8]. Magnetic bead cell sorting was employed to eliminate dead cells and enrich for the persisting cell population that survived the CRT treatment regimen (Fig. 1A). These persisting cells were re-challenged with RT (2 Gy) or high-dose chemotherapy (500 nM cisplatin or 1 nM vincristine) and showed markedly reduced sensitivity compared to therapy-naïve cells, confirming their CRT-resistant (CRT-Res) phenotype (Fig. 1B). To assess changes in kinase signaling pathways associated with therapy resistance, the Proteome Profiler Phospho-Kinase Array multiplex immunoassay kit was used to evaluate the phosphorylation of 37 kinases in therapy-naïve and CRT-Res SU_MB002 cells (Supplementary Fig. 1A). This comprehensive screening revealed increased phosphorylation of the SRC kinase at the activating tyrosine 419 (p-SRC Y419) site, as one of the top hits in CRT-Res G3 MB cells. Other kinases that exhibited elevated phosphorylation in CRT-Res cells included known downstream targets of SRC signaling, p-Lyn, p-PYK2, and p-PLC-γ1 [41–43] (Fig. 1C, D and Supplementary Fig. 2A). Interestingly, other kinases commonly implicated in oncogenesis, including p-AKT, p-ERK1/2, and p-JNK [19, 44] and total phosphorylated tyrosine (p-tyrosine) levels showed no significant changes post-CRT treatment (Fig. 1C, D and Supplementary Fig. 2B), suggesting tightly regulated alterations in phospho-kinase signaling following CRT, with SRC activation as a potential key mediator of therapy resistance in G3 MB. These findings were corroborated across all three G3 MB cell lines (Fig. 1E), demonstrating that this increase in SRC activation following CRT treatment is a conserved response in G3 MB, potentially mediating therapy resistance and tumor recurrence.

### Relapsed G3 MB tumors exhibit higher levels of SRC phosphorylation in vivo

**Fig. 2:**
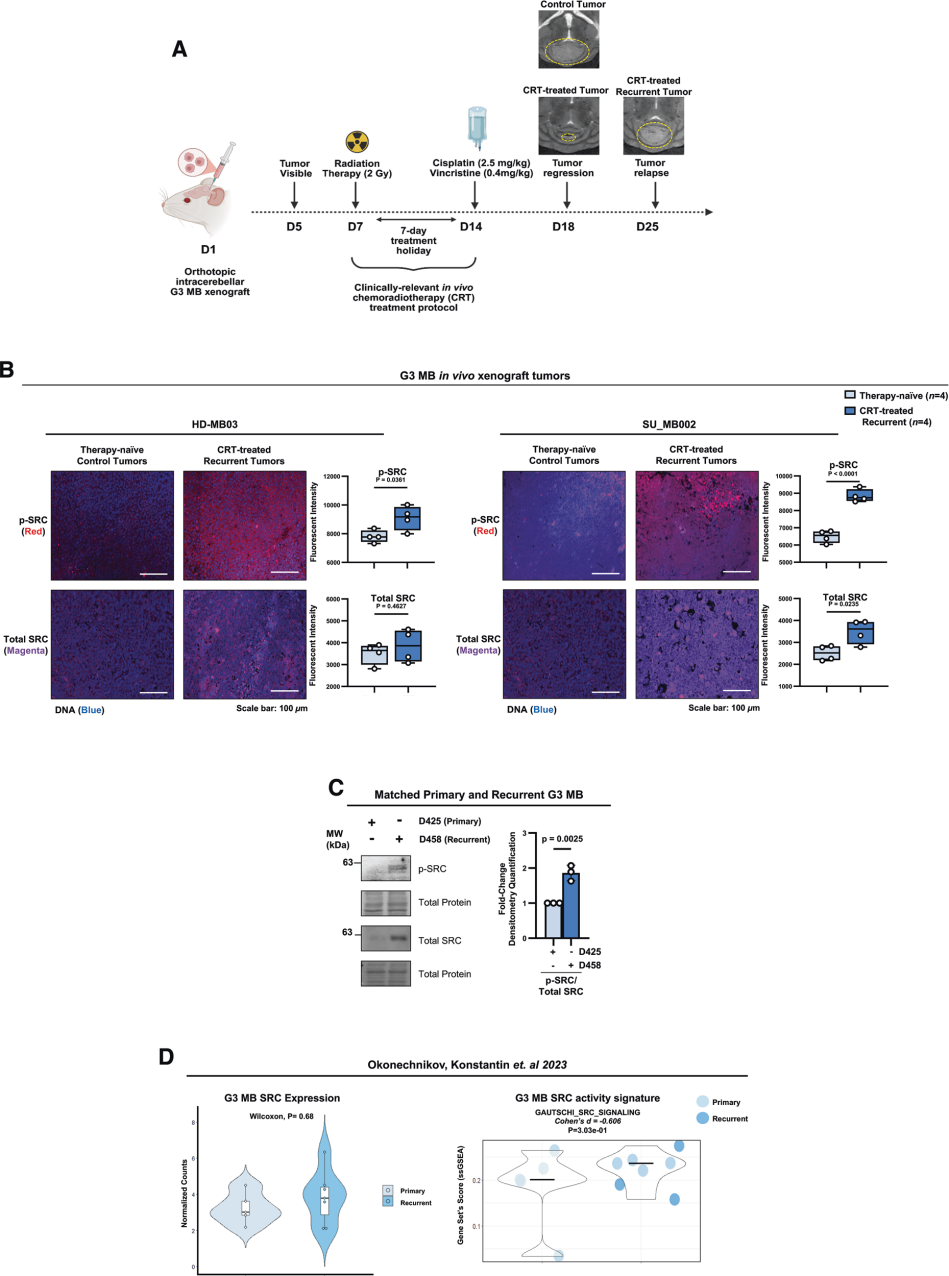
Relapsed G3 MB tumors exhibit higher levels of SRC phosphorylation in vivo*.* **A** Schematic diagram depicting the in vivo CRT treatment regimen, generated using Biorender**. B** Representative IF images of p-SRC (Y419) (red), total SRC (magenta), and DAPI-stained DNA (blue) in HD-MB03 and SU_MB002 therapy-naïve control tumors and CRT-treated recurrent tumors. Box-and-whisker plots represent quantification of fluorescent intensity from *n* = 4 tumor samples per group, with a solid line at the mean, unpaired two-tailed t test. **C** p-SRC (Y419) and total SRC immunoblots in CRT-treated matched primary (D425) and recurrent (458) G3 MB cells. Graph represents densitometry quantification measurement normalized to the total protein stain of p-SRC/total SRC from *n* = 3 experimental replicates presented as mean + s.e.m; unpaired two-tailed t tests. **D** Violin plot representing *SRC* expression and SRC activity signature abundance based on the GAUTSCHI_SRC_SIGNALING gene set in the data set from Okonechnikov K. et al. [40]. The Wilcoxon test was used for statistical analysis, and Cohen’s *d* was used to measure effect size.

To assess whether these signaling alterations represent transient changes induced by acute therapeutic stress in vitro or a long-term adaptation, sustained in recurrent tumors in vivo, we utilized two patient-derived orthotopic xenograft (PDOX) models, where SU_MB002 and HD-MB03 cells were stereotactically injected into the cerebellum of NSG mice [45]. Animals were subjected to an in vivo adapted CRT regimen [46], consisting of craniospinal RT (2 Gy) followed by treatment with cisplatin (2.5 mg/kg) and vincristine (0.4 mg/kg) (Fig. 2A). While CRT treatment initially induced tumor regression compared to untreated therapy-naïve controls, large recurrent tumors eventually formed in CRT-treated mice, mimicking disease relapse (Fig. 2A). Immunofluorescence (IF) analysis revealed that levels of p-SRC were significantly elevated in the recurrent tumors from CRT-treated mice in both models compared to treatment-naïve controls (Fig. 2B), supporting our in vitro findings and pinpointing SRC activation as a persistent adaptation to CRT in G3 MB.

To further establish the clinical relevance of our findings, we evaluated SRC activation in primary (D425) and relapsed (D458) G3 MB [32] samples obtained from the same patient, and observed drastically increased levels of total and p-SRC in recurrent D458 G3 MB (Fig. 2C). Additionally, we analyzed a publicly available gene expression dataset featuring paired primary and recurrent G3 MB samples [40]. Although total *SRC* mRNA was not significantly elevated, the SRC activity signature was markedly upregulated in recurrent G3 MB patient samples (Fig. 2D). Collectively, these data indicate that upregulation of p-SRC following CRT treatment is a clinically-relevant adaptation potentially driving G3 MB relapse.

### CRT-mediated activation of SRC is a G3 MB tumor- and subgroup-specific response

**Fig. 3:**
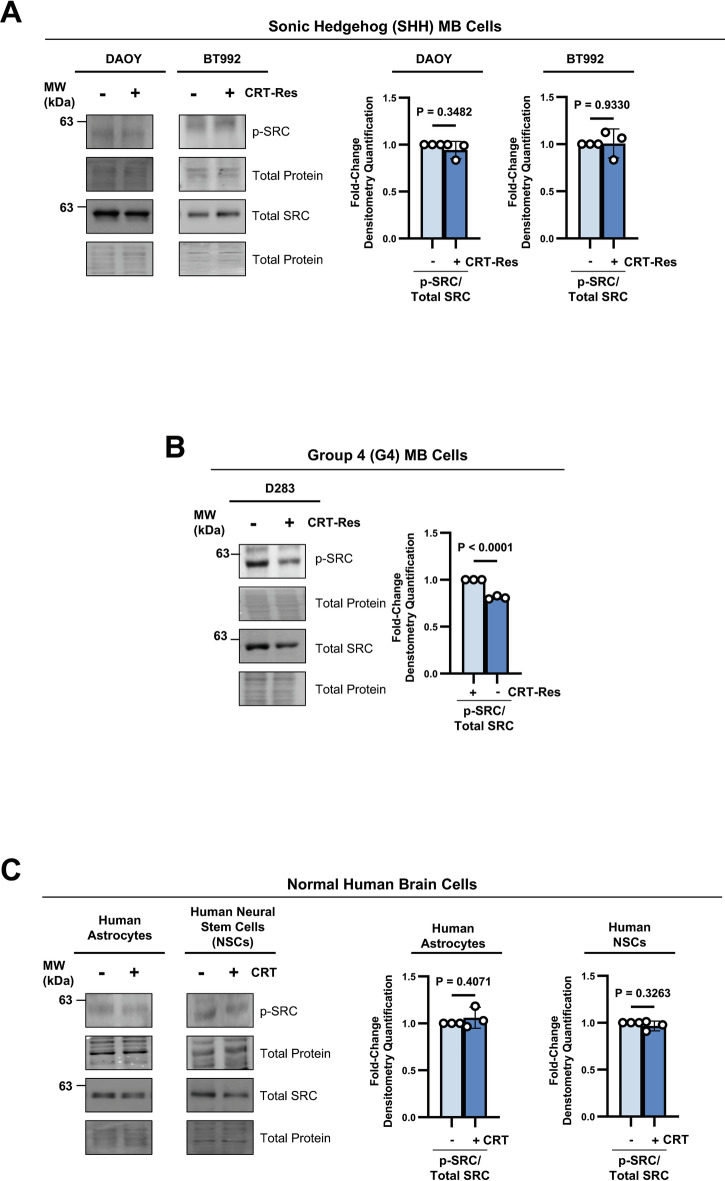
CRT-mediated activation of SRC is a G3 MB tumor- and subgroup-specific response. p-SRC (Y419) and total SRC immunoblots in CRT-treated **A** SHH MB cells (DAOY and BT992), **B** G3/G4 MB cells (D283), and **C** normal human brain cells (human astrocytes and human NSCs). Graphs represent densitometry quantification measurement normalized to the total protein stain of p-SRC/total SRC from *n* = 3 experimental replicates presented as mean + s.e.m; unpaired two-tailed t tests.

Moving forward, we wished to determine whether SRC activation is specific to G3 MB. SHH MB tumors typically exhibit better response to standard CRT, with lower relapse frequency and better prognosis [11, 12]. To assess whether subgroups with a favorable prognosis exhibit similar response to CRT, we utilized the well-established SHH cell line DAOY and the patient-derived SHH MB cell line BT992 [34, 35]. In contrast to G3 MB, SHH cells that survived CRT treatment exhibited no p-SRC upregulation (Fig. 3A).

As elevated SRC levels are a reported hallmark of G4 MB [47], we evaluated CRT-mediated changes in SRC activity in the D283 MB cell line, which has previously been classified as G4 [33, 48]. Interestingly, G4 MB cells responded to CRT treatment with a significant decrease in total and p-SRC levels (Fig. 3B), implying a mechanism of adaptation different from G3 MB. This suggests that activation of SRC may be a resistance mechanism restricted to the more aggressive G3 MB subgroup, contributing to their worse prognosis.

Our findings demonstrate that activation of SRC following CRT treatment is a persistent and conserved adaptation in G3 MB that may represent a potential therapeutic vulnerability. However, it is important to ensure that normal brain cells, particularly astrocytes and NSCs, do not use the same CRT-survival mechanisms, as damage to them is at the root of long-term health issues in many MB survivors [8, 9, 49]. Therefore, we evaluated p-SRC levels in normal human astrocytes and human NSCs post-CRT treatment. Importantly, we found that normal human brain cells did not exhibit p-SRC upregulation following CRT (Fig. 3C), suggesting that this signaling alteration may represent a tumor-specific adaptation. Overall, these results highlight CRT-mediated SRC activation as a potential therapeutic target that could selectively suppress recurrent G3 MB tumors while sparing normal cells.

### SRC activation is essential to maintain stemness in therapy-resistant G3 MB cells

Our findings thus far demonstrate that SRC is specifically activated in G3 MB cells following CRT treatment and persists in recurrent tumors. Next, we wished to understand the molecular and phenotypic role of SRC activation in CRT-Res G3 MB cells. SRC is known to regulate many cellular processes, including stemness [26, 50], which is in turn associated with aggressive tumor features, including relapse, metastasis, and poor prognosis [30, 51, 52]. Therefore, we investigated whether SRC regulates the stemness properties of G3 MB cells following CRT treatment.

**Fig. 4:**
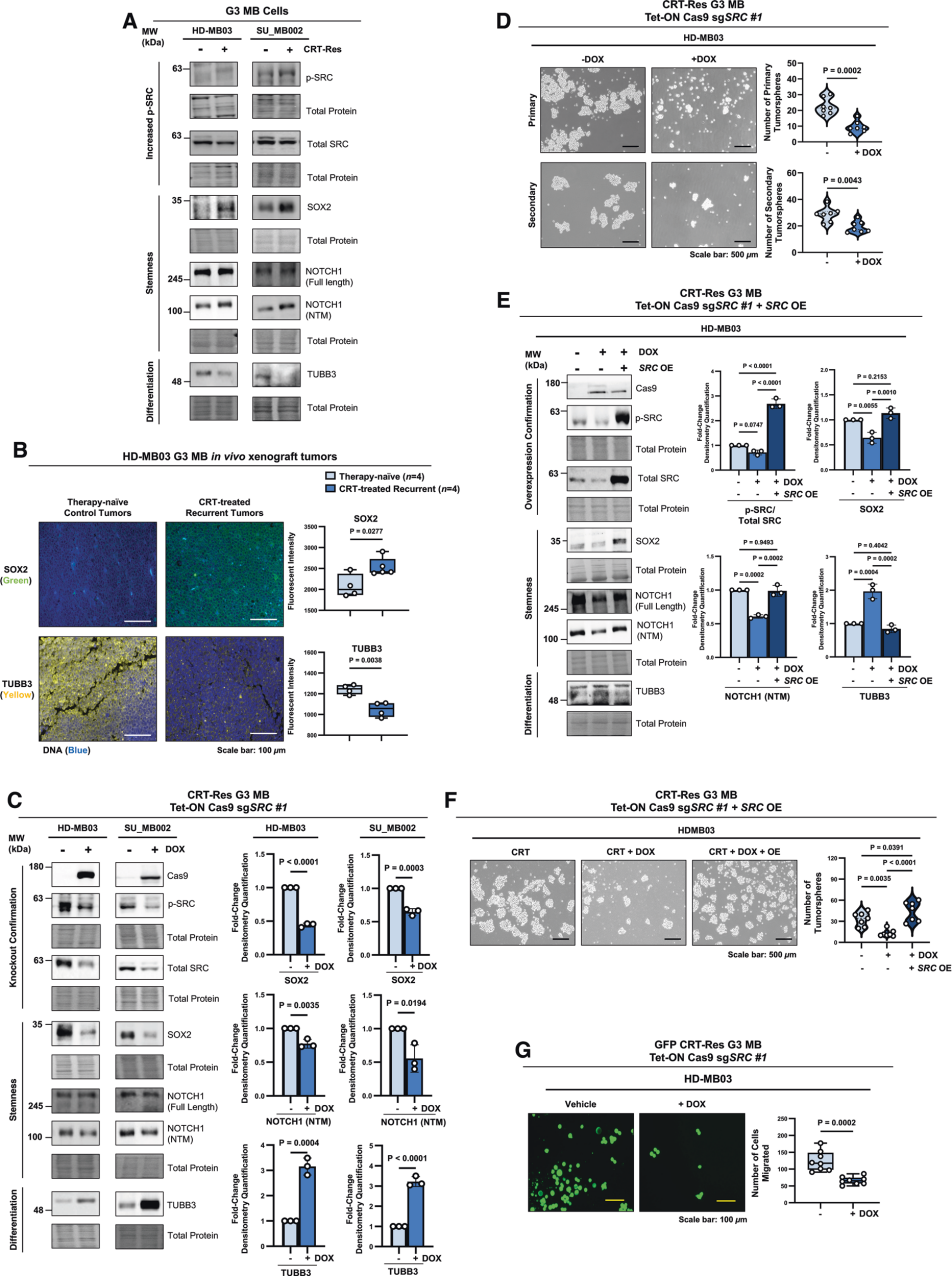
SRC activation is essential to maintain stemness in therapy-resistant G3 MB cells. **A** Immunoblot of p-SRC (Y419), total SRC, SOX2, NOTCH1 (full length and NTM), and TUBB3 expression in CRT-Res HD-MB03 and SU_MB002 cells. **B** IF images of HD-MB03 tumors from therapy-naïve and CRT-treated recurrent tumors stained with SOX2 (green) and TUBB3 (yellow) with DAPI (blue). Box-and-whisker plots represent quantification of fluorescent intensity from *n* = 3 tumor samples per group, with a solid line at the mean, unpaired two-tailed t test. **C** Immunoblots of Cas9, p-SRC (Y419), total SRC, SOX2, NOTCH1 (full length and NTM), and TUBB3 in CRT-Res HD-MB03 and SU_MB002 cells expressing Tet-ON Cas9 sg*SRC #1* treated with DOX to induce *SRC* KO. Graphs represent densitometry quantification measurement of SOX2, NOTCH1 (NTM), and TUBB3, normalized to the total protein stain levels from *n* = 3 experimental replicates, presented as mean ± s.e.m; unpaired two-tailed t test. **D** Primary and secondary tumorsphere formation assay from CRT-Res HD-MB03 cells expressing Tet-ON Cas9 sg*SRC* #1 treated with DOX to induce *SRC* KO. A violin plot represents the quantification of the total sphere number from *n* = 7 replicates, with dashed lines at the mean and quartiles; an unpaired two-tailed t-test. **E** Immunoblots of Cas9, p-SRC (Y419), total SRC, SOX2, NOTCH1 (full length and NTM), and TUBB3 in CRT-Res HD-MB03 cells expressing Tet-ON Cas9 sg*SRC #1* treated with DOX and transfected with *SRC* OE. Graphs represent densitometry quantifications of p-SRC/total SRC, SOX2, NOTCH1 (NTM), and TUB33 levels from *n* = 3 experiments; mean + s.e.m. two-way ANOVA with Tukey’s test. **F** Tumorsphere formation assay from CRT-Res HD-MB03 expressing Tet-ON Cas9 sg*SRC* #1 treated with DOX and transfected with *SRC* OE, Violin plot represents quantification of total sphere number from *n* = 7 replicates, with dashed lines at the mean and quartiles; two-way ANOVA with Tukey’s test. **G** Representative images and quantification of migration assays in HD-MB03 cells expressing Tet-ON Cas9, sg*SRC* #1, and GFP, treated with DOX to induce *SRC* KO. Box-and-whisker plots represent quantification of migrated cells from *n* = 8 replicates, with a solid line at the mean, and an unpaired two-tailed t-test.

Profiling of naïve and CRT-Res SU_MB002 and HD-MB03 cells showed a significant increase in stemness markers following CRT, including SRY-box transcription factor 2 (SOX2) [53], octamer-binding transcription factor 4 (OCT4) [54], phosphorylated signal transducer and activator of transcription 3 at tyrosine 705 (p-STAT3 Tyr705) [55], and homeobox protein NANOG [56] (Fig. 4A and Supplementary Fig. 3A, B). Additionally, we observed stemness-associated activation of the neurogenic locus notch homolog protein 1 (NOTCH1), evident from increased NOTCH1 cleavage into its ligand-binding extracellular (NEC) and transmembrane/cytoplasmic (NTM) domains [57–59] (Fig. 4A and Supplementary Fig. 3A). Furthermore, we observed marked loss of the neuronal differentiation marker βIII-tubulin (TUBB3), indicating a transition towards a less differentiated state (Fig. 4A and Supplementary Fig. 3A). Importantly, IF showed an increase in the stemness factor SOX2 and decreased TUBB3 in both SU_MB002 and HD-MB03 recurrent PDOX tumors (Fig. 4B and Supplementary Fig. 3C). These observations suggest that CRT-Res G3 MB have enhanced stem-like properties both in vitro and in vivo.

To further ascertain if SRC helps maintain the enhanced stemness properties of CRT-Res G3 MB cells, we developed a Tet-ON Cas9 *SRC* knock-out (KO) system in HD-MB03 and SU_MB002 cells using two distinct *SRC* gRNA (sg*SRC*) clones. Following CRT treatment, G3 MB cells expressing Tet-ON Cas9 and sg*SRC* were sorted to isolate living cells and subsequently treated with DOX for 3 days to induce Cas9 expression and *SRC* KO, which also diminished p-SRC and total p-tyrosine levels consistent with kinase loss-of-function (Fig. 4C and Supplementary Fig. 4A). We found that loss of *SRC* corresponded with a marked reduction in SOX2, OCT4, p-STAT3, NANOG, and NOTCH1 cleavage, which was accompanied by an increase in TUBB3 [60] (Fig. 4C and Supplementary Fig. 4B, C), indicating a shift towards a more differentiated phenotype. Moreover, functional assessment of stem cell self-renewal capacity using the gold-standard tumorsphere formation assay [61] showed a decrease in the number of primary and secondary tumorspheres following *SRC* KO, confirming loss of stemness characteristics (Fig. 4D and Supplementary Fig. 4D, E). Importantly, restoring active *SRC* expression via gain-of-function OE rescued levels of stemness markers and tumorsphere formation capacity, while suppressing TUBB3 expression in *SRC* KO CRT-Res G3 MB cells (Fig. 4E, F), highlighting the critical role of SRC in directly regulating stemness in CRT-treated G3 MB.

Given the high metastatic incidence in recurrent G3 MB and the link between stemness and invasiveness [11, 12, 59, 62], we investigated the role of SRC on metastatic potential using the transwell migration assay. In line with the loss of stemness, we found that *SRC* KO also significantly reduced the migratory capacity of CRT-Res HD-MB03 and SU_MB002 G3 MB cells (Fig. 4G and Supplementary Fig. 4F). Taken together, these results demonstrate that SRC actively contributes to stem-like and aggressive migratory phenotypes of therapy-resistant G3 MB cells.

### Loss of SRC leads to cell death in therapy-resistant G3 MB cells through the activation of apoptosis and necroptosis

**Fig. 5:**
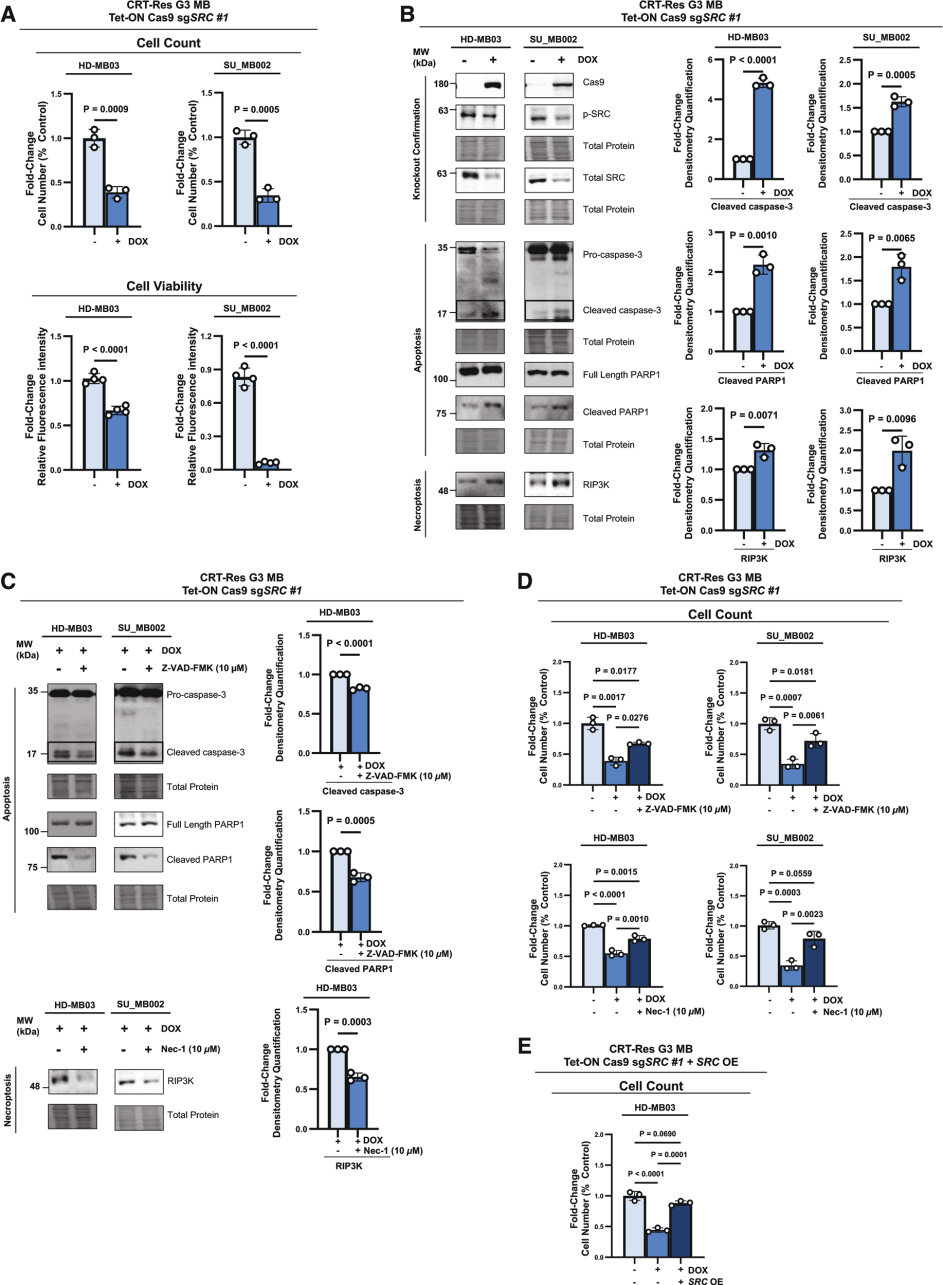
Loss of SRC leads to cell death in therapy-resistant G3 MB cells through the activation of apoptosis and necroptosis. **A** Cell count (top panel) and viability PrestoBlue (bottom panel) analysis of CRT-Res HD-MB03 and SU_MB002 cells expressing Tet-ON Cas9 sg*SRC #1* treated with DOX to induce *SRC* KO. Graphs represent fold-change in cell number and fluorescent PrestoBlue signal in *SRC* KO cells (+DOX) compared to controls (-DOX). Data presented as mean ± s.e.m; from *n* = 3 (cell count) and *n* = 4 (cell viability) experimental replicates; unpaired two-tailed t-test. **B** Immunoblot of Cas9, p-SRC (Y419), total SRC, pro-caspase-3, cleaved caspase-3, full length PARP1, cleaved PARP1, and RIP3K in CRT-Res HD-MB03 and SU_MB002 cells expressing Tet-ON Cas9 sg*SRC #1* treated with DOX. Graphs represent densitometry quantification measurement of cleaved caspase-3, cleaved PARP1, and RIP3K normalized to the total protein stain levels from *n* = 3 experimental replicates, presented as mean ± s.e.m; unpaired two-tailed t test. **C** Immunoblot of pro-caspase-3, cleaved caspase-3, full-length PARP1, cleaved PARP1, and RIP3K in CRT-Res HD-MB03 and SU_MB002 cells expressing Tet-ON Cas9 sg*SRC* #1 following DOX-induced *SRC* KO treated with Z-VAD-FMK (10 µM) or Nec-1 (10 µM) treatment. Graphs represent densitometry quantification measurement stain of cleaved caspase-3, cleaved PARP1, and RIP3K normalized to the total protein levels in CRT-Res HDMB03and SU_MB002 cells from *n* = 3 experimental replicates, presented as mean ± s.e.m; unpaired two-tailed t test. **D** Cell count analysis of CRT-Res HD-MB03 and SU_MB002 cells expressing Tet-ON Cas9 sg*SRC* #1 treated with DOX and Z-VAD-FMK (10 µM) (top panel) and Nec-1 (10 µM) (bottom panel) treatment. Graphs represent fold change against CRT treatment alone from *n* = 3 experimental replicates, presented as mean + s.e.m; two-way ANOVA with Tukey’s test. **E** Cell count analysis of CRT-Res HD-MB03 cells expressing Tet-ON Cas9 sg*SRC* #1 treated with DOX and transfected with *SRC* OE. Graphs represent fold change against CRT treatment alone from *n* = 3 experimental, presented as mean + s.e.m; two-way ANOVA with Tukey’s test.

Our data highlights a pro-tumorigenic role of SRC in maintaining the aggressive stemness and migration characteristics of CRT-Res G3 MB cells. Although SRC is known to regulate both stemness and proliferation, its role is context-dependent, where SRC can promote both cell growth and cell death depending on the circumstances [28, 50, 63–66]. Therefore, we aimed to clarify the role of SRC in the viability of CRT-Res G3 MB cells. Using trypan blue exclusion and PrestoBlue viability assays, we found that *SRC* KO in CRT-Res HD-MB03 and SU_MB002 cells resulted in a significant reduction in cell number and viability (Fig. 5A and Supplementary Fig. 5A).

Further analysis demonstrated that *SRC* loss in CRT-Res HD-MB03 and SU_MB002 cells induced cell death *via* both apoptosis and necroptosis, as evident from activation of cleaved caspase-3 and its downstream target PARP-1, concurrent with upregulation of the necroptosis marker receptor-activated protein kinase 3 (RIP3K) (Fig. 5B and Supplementary Fig. 5B). To confirm that apoptosis and necroptosis are the primary modes of cell death following *SRC* loss, CRT-Res HD-MB03 and SU_MB002 *SRC* KO cells were treated with the pan-caspase apoptosis inhibitor, Z-VAD-FMK, or the necroptosis inhibitor, Nec-1. Z-VAD-FMK effectively inhibited caspase-3 and PARP-1 cleavage, while Nec-1 reduced RIP3K levels in CRT-Res *SRC* KO G3 MB cells, confirming disruption of respective pathways (Fig. 5C and Supplementary Fig. 5C). Each inhibitor partly restored the number of living cells in *SRC* KO cells, confirming that cell death following loss of *SRC* occurs *via* both apoptosis and necroptosis (Fig. 5D). Importantly, restoring active *SRC* expression in *SRC* KO CRT-Res G3 MB cells blocked the upregulation of pro-apoptosis and necroptosis markers and rescued cell numbers, highlighting that activation of these pathways is dependent on loss of *SRC* (Fig. 5E and Supplementary Fig. 5D).

Altogether, these findings underscore the important role of SRC in supporting the survival of CRT-Res G3 MB cells. Targeting this key signaling regulator induces cell death through multiple pathways, reducing the likelihood of G3 MB persistence.

### Pharmacological targeting of SRC in therapy-resistant G3 MB cells promotes cell death and differentiation

Our results highlight targeting SRC as a promising strategy to induce cell death and differentiation in CRT-Res G3 MB cells, suppressing their aggressive stemness and metastatic properties. This approach has immediate clinical potential, as several SRC inhibitors are FDA-approved or are undergoing clinical trials, but have not been tested in G3 MB, making them suitable candidates for drug repurposing. We tested two blood-brain-barrier (BBB) permeable SRC inhibitors: Bosutinib, which is FDA-approved for chronic myeloid leukemia, and Saracatinib, currently in clinical trials for idiopathic pulmonary fibrosis (NCT04598919) [23, 67]. Additionally, we included an experimental, yet potent and specific Src-family inhibitor, SU6656 [68].

**Fig. 6:**
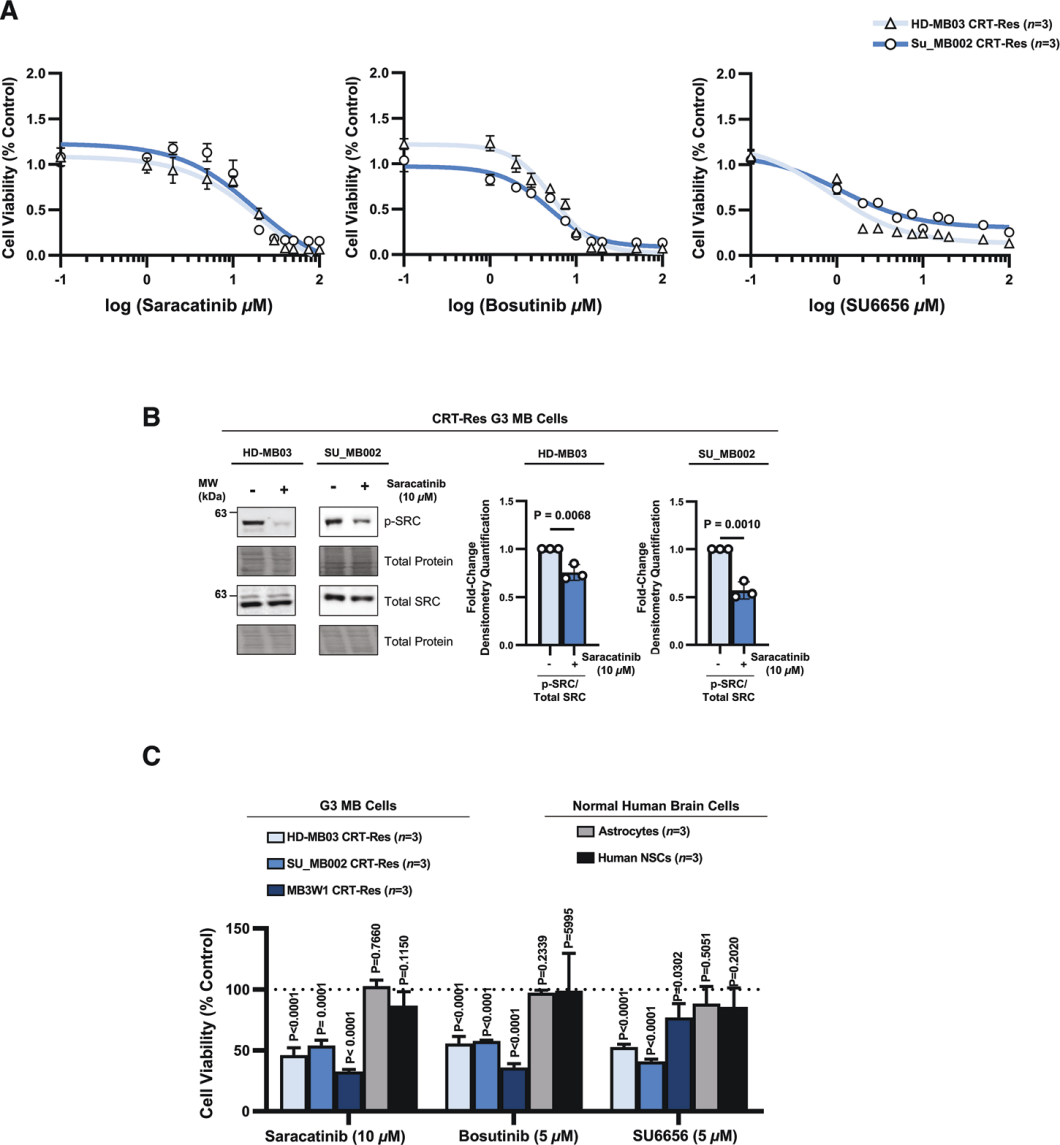
SRC inhibitors exhibit dose-dependent cytotoxicity in therapy-resistant G3 MB cells with minimal impact on normal human brain cells. **A** Dose-response curves of cell viability measured using PrestoBlue in CRT-Res HD-MB03 and SU_MB002 cells treated with increasing concentrations of Saracatinib, Bosutinib, or SU6656. Values plotted as mean % of vehicle-treated CRT-Res controls ±s.e.m. **B** Immunoblot of p-SRC (Y419) and total SRC in CRT-Res HD-MB03 and SU_MB002 cells treated with Saracatinib. Graphs represent densitometry quantification measurement of p-SRC/total SRC normalized to the total protein stain from *n* = 3 experiments, presented as mean ± s.e.m.; unpaired two-tailed t tests. **C** Cell viability measured using PrestoBlue in CRT-Res G3 MB cells (HD-MB03, SU_MB002, and MB3W1) and normal human brain cells (human astrocytes and human NSCs) treated with Saracatinib (10 µM), Bosutinib (5 µM), or SU6656 (5 µM). Fluorescent PrestoBlue signal calculated as % of vehicle-treated controls from *n* = 3 experimental replicates plotted as mean + s.e.m; using unpaired two-tailed t tests.

Dose-response analysis in CRT-Res SU_MB002 and HD-MB03 cells revealed significant sensitivity to all three inhibitors (Fig. 6A). Appropriate doses for each inhibitor were selected and confirmed to inhibit SRC phosphorylation and activation (Fig. 6B and Supplementary Fig. 6A). The selected doses were tested in combination with CRT across three G3 MB cell lines, as well as in normal brain cells. Results indicated that SRC inhibitors substantially reduced viability in CRT-Res G3 MB cells, but did not impact either human astrocytes or NSCs, suggesting a tumor-specific response and a favorable toxicity profile (Fig. 6C).

**Fig. 7:**
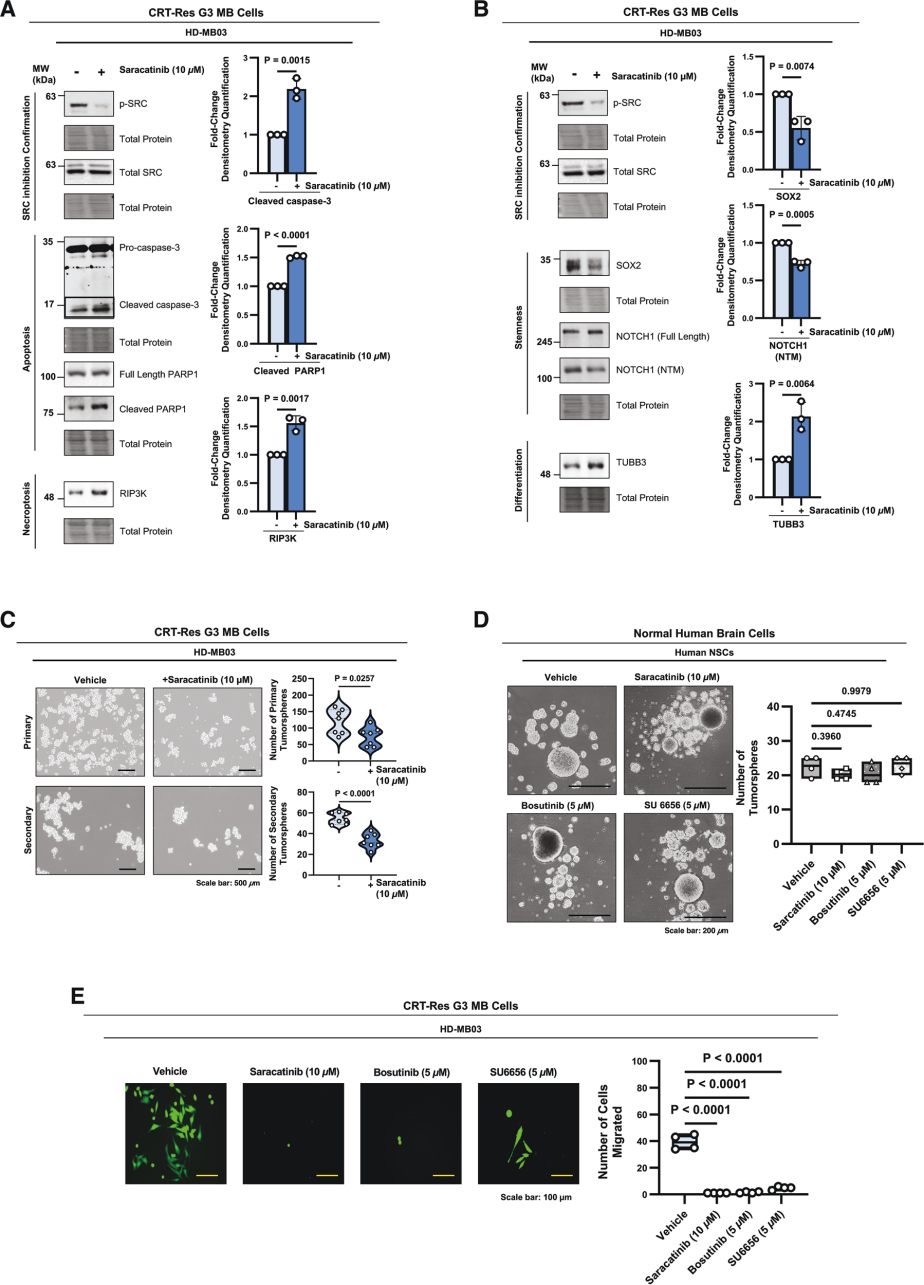
Pharmacological targeting of SRC in therapy-resistant G3 MB cells promotes cell death and differentiation. **A** Immunoblot of p-SRC (Y419), total SRC, pro-caspase-3, cleaved caspase-3, full length PARP1, cleaved PARP1, and RIP3K in CRT-Res HD-MB03 cells treated with Saracatinib (10 µM). Graphs represent densitometry quantification measurement normalized to cleaved caspase-3, cleaved PARP1, and RIP3K to the total protein stain levels from *n* = 3 experimental replicates, presented as mean ± s.e.m; unpaired two-tailed t test. **B** Protein blots of p-SRC (Y419), total SRC, SOX2, NOTCH1 (full length and NTM), and TUBB3 in CRT-Res HD-MB03 cells treated with Saracatinib (10 µM). Graphs represent densitometry quantification measurement of SOX2, NOTCH1 (NTM), and TUBB3 normalized to the total protein stain levels from *n* = 3 experimental replicates, presented as mean ± s.e.m; unpaired two-tailed t test. **C** Primary and secondary tumorsphere formation assay of CRT-Res HD-MB03 cells treated with Saracatinib (10 µM). A violin plot represents the quantification of the total sphere number from *n* = 7 replicates, with dashed lines at the mean and quartiles; an unpaired two-tailed t-test. **D** Sphere formation analysis of human NSCs treated with Saracatinib (10 µM), Bosutinib (5 µM), or SU6656 (5 µM). Box plot graphs represent quantification of the total sphere number from *n* = 4 replicates; one-way ANOVA with Dunnett’s test. **E** Representative images and quantification of migration assays in HD-MB03 cells treated with Saracatinib (10 µM), Bosutinib (5 µM), or SU6656 (5 µM). Box plot graphs represent quantification from *n* = 4 experimental replicates, with a solid line at the mean; two-way ANOVA with Tukey’s test.

In line with findings from *SRC* KO cells, inhibition of SRC with Saracatinib in CRT-Res HD-MB03 cells led to caspase-3 and PARP1 cleavage, as well as RIP3K accumulation (Fig. 7A), indicating that pharmacological inhibition induces cell death through both apoptosis and necroptosis. Moreover, Saracatinib reduced stemness and promoted differentiation, evidenced by decreased SOX2 levels and NOTCH1 activation alongside increased TUBB3 expression, and a reduction in the ability of CRT-Res cells to form primary and secondary tumorspheres (Fig. 7B, C and Supplementary Fig. 6B). Notably, stemness characteristics of NSCs remained unaffected by SRC inhibition, indicating that, unlike G3 MB, they do not rely on SRC to support self-renewal (Fig. 7D). These findings suggest that targeting p-SRC may provide a therapeutic benefit while limiting adverse effects on neurodevelopment in children. Furthermore, we found that all three SRC inhibitors reduced the migration of CRT-Res HD-MB03 cells (Fig. 7E), highlighting the potential to reduce the risk of distant metastases, which is a major risk factor for poor patient outcomes [10, 12]. Together, these results underscore SRC as a promising therapeutic target in recurrent G3 MB.

### Targeting p-SRC in recurrent G3 MB provides a survival benefit in vivo

**Fig. 8:**
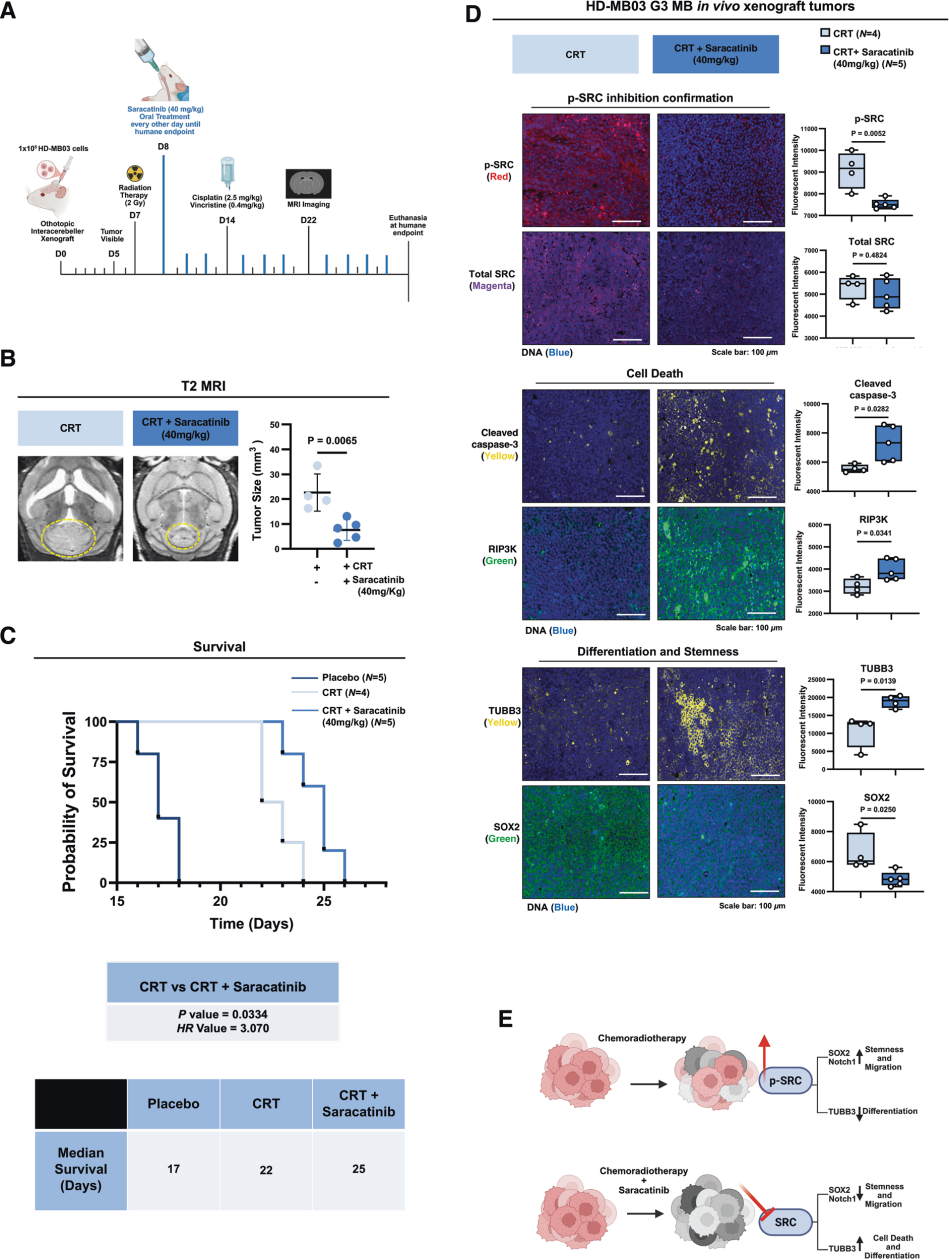
Targeting p-SRC in recurrent G3 MB provides a survival benefit in vivo. **A** Schematic of animal experiment design generated using Biorender. **B** Representative T2-weighted MRI images acquired 22 days post-transplant, with tumor margins outlined. Quantification of tumor volumes across treatment groups (*n* = 4 for CRT alone, *n* = 5 for CRT +Saracatinib); unpaired two-tailed t test. **C** Kaplan–Meier survival curves of mice treated with CRT alone or CRT + Saracatinib. P and HR values reflect the statistical comparison between the CRT alone and CRT + Saracatinib group, calculated using the log-rank method. The table represents the median survival in days of the Placebo, CRT, and CRT+ Saracatinib groups. **D** IF images of xenograft tumors from CRT-treated and CRT + Saracatinib-treated recurrent tumors labeled with (top) p-SRC (red) and total SRC (magenta); (middle) cleaved caspase-3 (yellow) and RIP3K (green); (bottom) TUBB3 (yellow) and SOX2 (green), and DAPI (blue). Graphs show quantification of fluorescence intensity from *n* = 4 (CRT alone) and *n* = 5 (CRT + Saracatinib) animals per group, with solid lines at the mean; unpaired two-tailed t test. **E** Summary diagram illustrating the effect of CRT with or without Saracatinib, generated using Biorender.

To evaluate SRC inhibition as a potential therapeutic strategy against recurrent G3 MB cells under physiologically relevant conditions, the established HD-MB03 PDOX G3 MB model was utilized, and animals were treated with a combination treatment regimen consisting of CRT supplemented with the BBB-permeable SRC inhibitor, Saracatinib. Following initial RT treatment, animals were randomly assigned to receive either 40 mg/kg Saracatinib or methylcellulose suspension placebo control every other day (QOD) by oral gavage (Fig. 8A). MRI indicated that while animals receiving CRT alone developed considerable recurrent tumors, the combination treatment with Saracatinib significantly reduced tumor burden (Fig. 8B and Supplementary Fig. 7A).

Importantly, in this aggressive in vivo G3 MB model, combinatorial treatment extended median survival time by approximately 47% compared to placebo controls (Fig. 8C). While multi-modal CRT treatment prolongs the median survival of animals by only 5.5 days compared to the placebo, combining of CRT with Saracatinib provided 8-day survival benefit, representing an additional ~15% increase (HR: 3.070) (Fig. 8C). IF profiling of tissues confirmed p-SRC depletion in the Saracatinib treated group, confirming on-target effects. Evaluation of cleaved caspase-3 and RIP3K levels indicated that p-SRC inhibition led to the induction of apoptosis and necroptosis in recurrent G3 MB tumors in vivo (Fig. 8D). Moreover, reduction in SOX2 expression, accompanied by TUBB3 upregulation in the combinatorial treatment group, suggests an increase in differentiation (Fig. 8D). Additionally, we evaluated treatment neurotoxicity using IBA1 as a marker of microglia activation and Bielschowsky silver staining to monitor neuron health. IBA1 IF revealed a mild decrease in active microglia following CRT compared to placebo, but combination with Saracatinib did not exacerbate this effect (Supplementary Fig. 7B). Furthermore, silver staining showed no changes in optical density or neuron fiber morphology with CRT or combinatorial treatment compared to placebo controls (Supplementary Fig. 7C). In summary, these findings suggest that p-SRC upregulation is a contributing factor to G3 MB recurrence, promoting stemness and survival mechanisms. Blocking SRC activation following CRT treatment is associated with increased differentiation and the induction of cell death, without causing additional toxicity (Fig. 8E).

## Discussion

MB is the most common malignant pediatric brain tumor, with G3 MB being the most aggressive subgroup [69]. Approximately 30% of G3 MB patients experience tumor relapse, with recurrent tumors refractory to standard therapy and lacking effective treatment options [12, 49].

The development of novel therapeutic strategies is hindered by the lack of matched primary-recurrent samples for research into MB recurrence. While gene expression datasets of recurrent MB are emerging [40], they do not fully recapitulate signaling changes at the protein level. To address this, we used three well-established G3 MB cell models and subjected them to in vitro and in vivo CRT regimens resembling standard care. Use of patient-derived cell lines has inherent limitations, including phenotypic drift and altered drug response associated with 2D monolayer culture [70–72]. However, these effects can be mitigated by using multiple distinct patient-derived samples, 3D suspension culture systems, and PDOX tissue profiling, allowing assessment of both acute and long-term treatment-induced adaptations [70, 73]. Profiling post-CRT adaptations revealed reduced differentiation and increased stemness markers, including NOTCH1 activation and upregulation of SOX2, consistent with previous reports that NOTCH1 and SOX2 can promote the transcription of each other to form a positive feedback loop driving stemness [57, 59]. These data also support previous clinical observations that increased cellular plasticity and metastasis frequently accompany G3 MB relapse [12, 13]. Despite the noticeable phenotypical changes, G3 MB tumors tend to retain subgroup-specific genetic makeup upon relapse, suggesting non-genetic drivers of treatment resistance and aggressiveness [13].

Kinases, key signaling mediators of cellular responses, can drive oncogenesis and have proven to be excellent targets for both monotherapy and adjuvant treatment in multiple cancer types [17, 18, 20, 74–78]. Yet, their role in resistance to standard chemotherapy in G3 MB remains poorly understood. Kinase profiling revealed that CRT treatment increases SRC phosphorylation specifically in G3 MB but not in normal brain cells or in less aggressive SHH and G4 MB subgroups, despite G4 MB having high basal levels of SRC expression [47]. While the SRC inhibitor dasatinib has been shown to suppress metastasis in treatment-naïve G3 MB in vivo, the role of SRC in G3 MB recurrence remains unexplored [79]. SRC plays a versatile, context-dependent role in both normal and cancer cells [16, 80]. SRC regulates diverse pathways, including migration and metabolism-fueled proliferation, but can also promote cell death through direct caspase activation or pro-inflammatory mediators, highlighting its dual pro- and anti-tumorigenic functions [24, 66, 77, 81]. In this study, we show that KO or inhibition of SRC induced both apoptosis and necroptosis in CRT-Res cells. These findings provide mechanistic insight into the role of SRC in recurrent G3 MB and suggest targeting SRC as a promising therapeutic strategy, as triggering two independent cell death pathways may yield a better response against heterogeneous tumor populations.

Tumor heterogeneity poses a significant challenge to the success of treatment; thus, therapies that also induce differentiation could improve outcomes [8, 13, 52, 82, 83]. Importantly, the small population of G3 MB cells that withstood combined CRT and SRC inhibition showed reduced stemness and invasiveness, suggesting that remnant cells became less aggressive. Consequently, in vivo combination treatment with SRC inhibitor Saracatinib and CRT reduced tumor burden and improved survival compared to CRT alone. Tissue profiling confirmed that this treatment induced differentiation and cell death through apoptosis and necroptosis.

Furthermore, Saracatinib has shown a favorable toxicity profile, sparing normal human brain cells and showing no significant neurotoxicity in vivo. Our findings align with data from phase I/II clinical trials for pulmonary fibrosis and Alzheimer’s disease, which indicate that Saracatinib is well-tolerated [23]. This positions Saracatinib as an excellent candidate for drug repurposing to target SRC activation in recurrent G3 MB. This combinatorial strategy may enhance treatment efficacy and improve outcomes for children facing G3 MB recurrence.

## Supplementary information

Supplementary Information

Supplementary Table 1

Supplementary Table 2

Uncropped Western Blots

## Data Availability

All data generated or analyzed during this study are included in this manuscript and its supplementary information files. Original uncropped Western blot images are provided in the supplementary files. Tables with reagents and antibodies used (Supplementary Table 1 and Supplementary Table 2) are available in the supplementary files.
