# Supplementary material for: Targeting SRC enhances differentiation and promotes multifaceted cell death mechanisms in recurrent group 3 medulloblastoma
Source: Cell Death Dis. 2026 Apr 24;17(1):543. doi: 10.1038/s41419-026-08751-9 (PMC13237022; doi:10.1038/s41419-026-08751-9)
Supplement: Supplementary file 1 — Supplementary Information [file 41419_2026_8751_MOESM1_ESM.pdf]

**SUPPLEMENTARY INFORMATION**

# Targeting SRC enhances differentiation and promotes multifaceted cell death mechanisms in recurrent group 3 medulloblastoma

Kuzmychova H., Chawla U. & Martell E. *et al*

# Supplementary Figure 1

## A

**Proteome Profiler Phospho-Kinase Array**

**Membrane layout and blot coordinates**

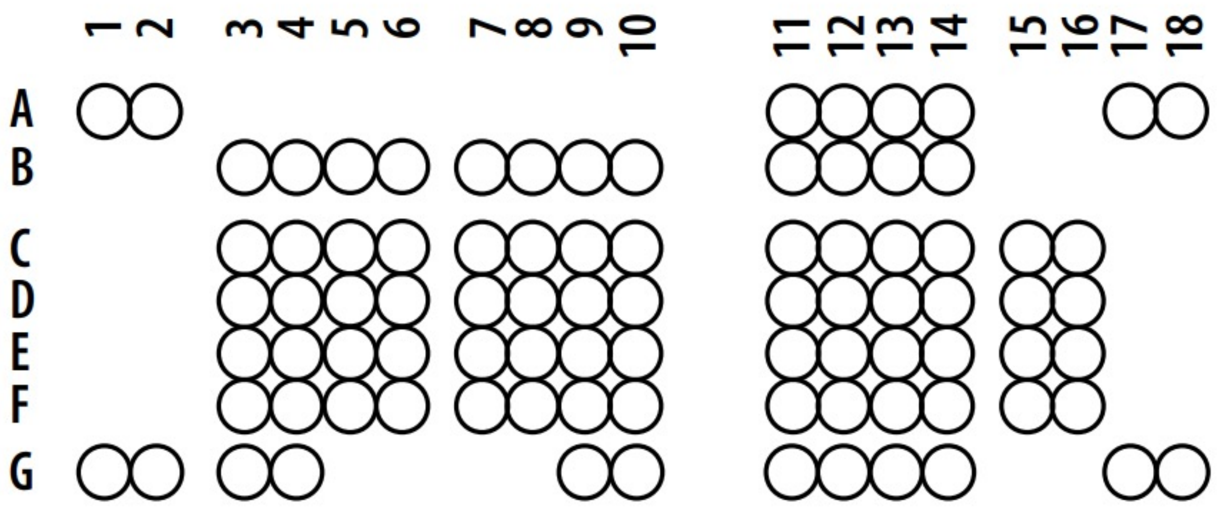


| Coordinates | Target/Control | Phosphorylation Site |
|---|---|---|
| A1,A2 | Reference Spot | ________ |
| A11,A12 | Akt 1/2/3 | T308 |
| A13, A14 | Akt 1/2/3 | S473 |
| A17, A18 | Reference Spot | ________ |
| B3, B4 | CREB | S133 |
| B5, B6 | EGF R | Y1086 |
| B7, B8 | eNOS | S1177 |
| B9,B10 | ERK1/2 | T202/Y204,<br>T185/Y187 |
| B11, B12 | Chk-2 | T68 |
| B13,B14 | c-Jun | S63 |
| C3,C4 | Fgr | Y412 |
| C5,C6 | GSK-3α/β | S21/S9 |
| C7,C8 | GSK-3β | S9 |
| C9, C10 | HSP27 | S78/S82 |
| C11,C12 | p53 | S15 |
| C13, C14 | p53 | S46 |
| C15, C16 | p53 | S392 |
| D3, D4 | JNK 1/2/3 | T183/Y185,<br>T221/T223 |
| D5, D6 | Lck | Y394 |
| D7, D8 | Lyn | Y397 |
| D9, D10 | MSK1/2 | S376/S360 |
| D11, D12 | P70 S6 Kinase | T389 |
| D13, D14 | P70 S6 Kinase | T421/S424 |
| D15, D16 | PRAS40 | T246 |

| Coordinates | Target/Control | Phosphorylation Site |
|---|---|---|
| E3, E4 | p38α | T180/Y182 |
| E5, E6 | PDGF Rβ | Y7751 |
| E7, E8 | PLC-γ1 | Y783 |
| E9, E10 | SRC | Y419 |
| E11, E12 | PYK2 | Y402 |
| E13, E14 | RSK1/2 | S221/S227 |
| E15, E16 | RSK1/2/3 | S380/S386/S377 |
| F3, F4 | STAT2 | (Py690) |
| F5, F6 | STAT5a/b | Y694/y699 |
| F7, F8 | WNK1 | T60 |
| F9, F10 | Yes | Y426 |
| F11, F12 | STAT1 | Y701 |
| F13, F14 | STAT3 | Y705 |
| F15, F16 | STAT3 | S727 |
| G1, G2 | Reference Spot | ________ |
| G3, G4 | B-Catenin | ________ |
| G9, G10 | PBS (Negative Control) | ________ |
| G11, G12 | STAT6 | |
| G13, G14 | HSP60 | ________ |
| G17, G18 | PBS (Negative Control) | ________ |

**Supplementary Figure 1**. **(A)** Membrane layout and blot coordinates of proteome profile phospho-kinase array.

# Supplementary Figure 2

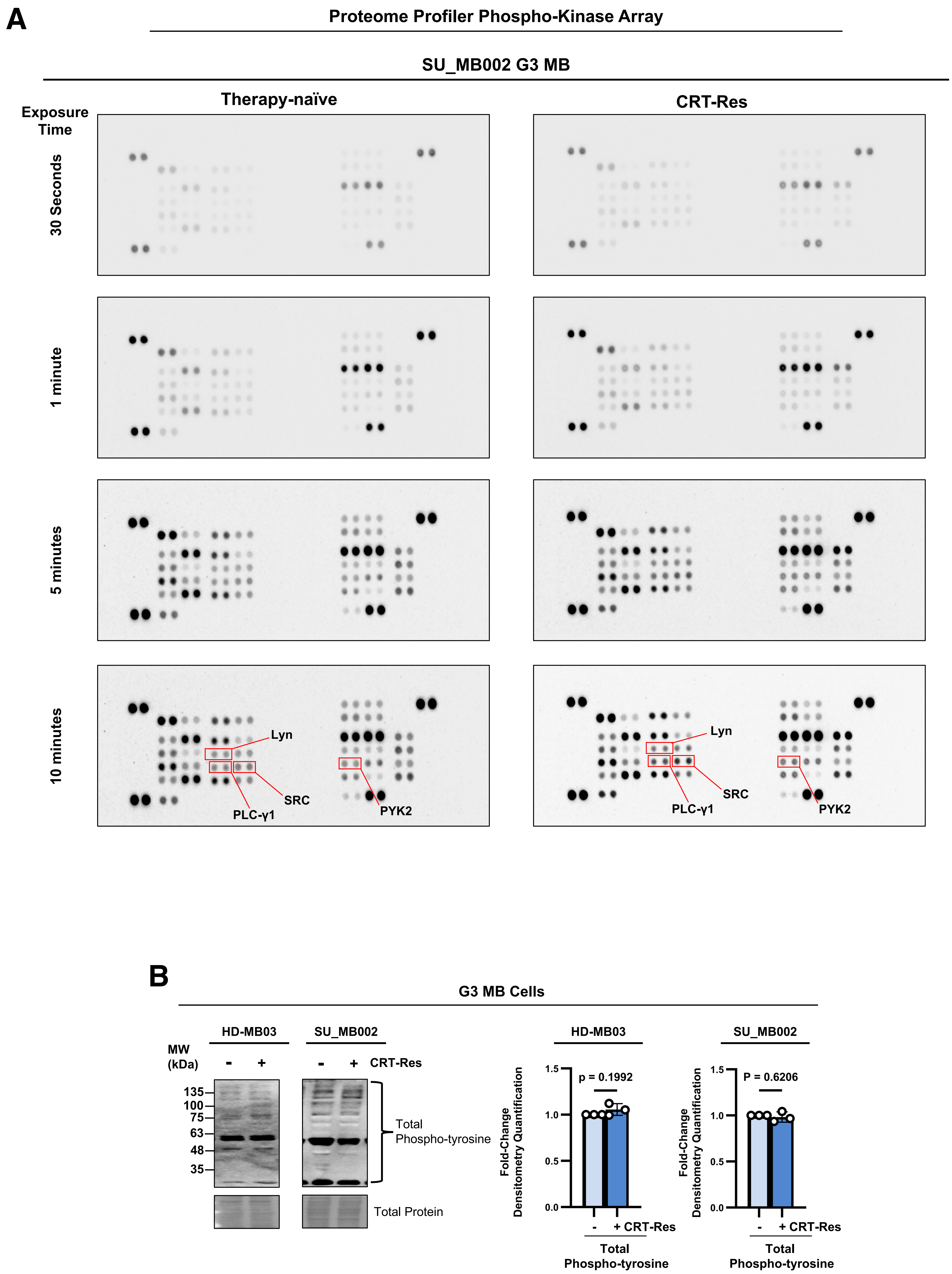


**Supplementary Figure 2**. **(A)** Uncut Phospho-kinase array blots of therapy naïve and CRT-Res SU_MB002 samples. Array blots imaged at multiple exposure times (30 seconds, 1 minute, 5 minutes, and 10 minutes). Red boxes highlight the locations of the kinases of interest presented in Figure 1D (p-SRC, p-Lyn, p-PYK2, p-PLC-γ1, p-AKT, p-ERK, and p-JNK). **(B)** Anti-Phospho tyrosine levels in CRT-Res HD-MB03 and SU_MB002 cells. Graphs represent densitometry quantification measurement of Anti-Phospho Tyrosine normalized to the total protein from $n = 3$ experimental replicates, presented as mean ± s.e.m; unpaired two-tailed t test.

# Supplementary Figure 3

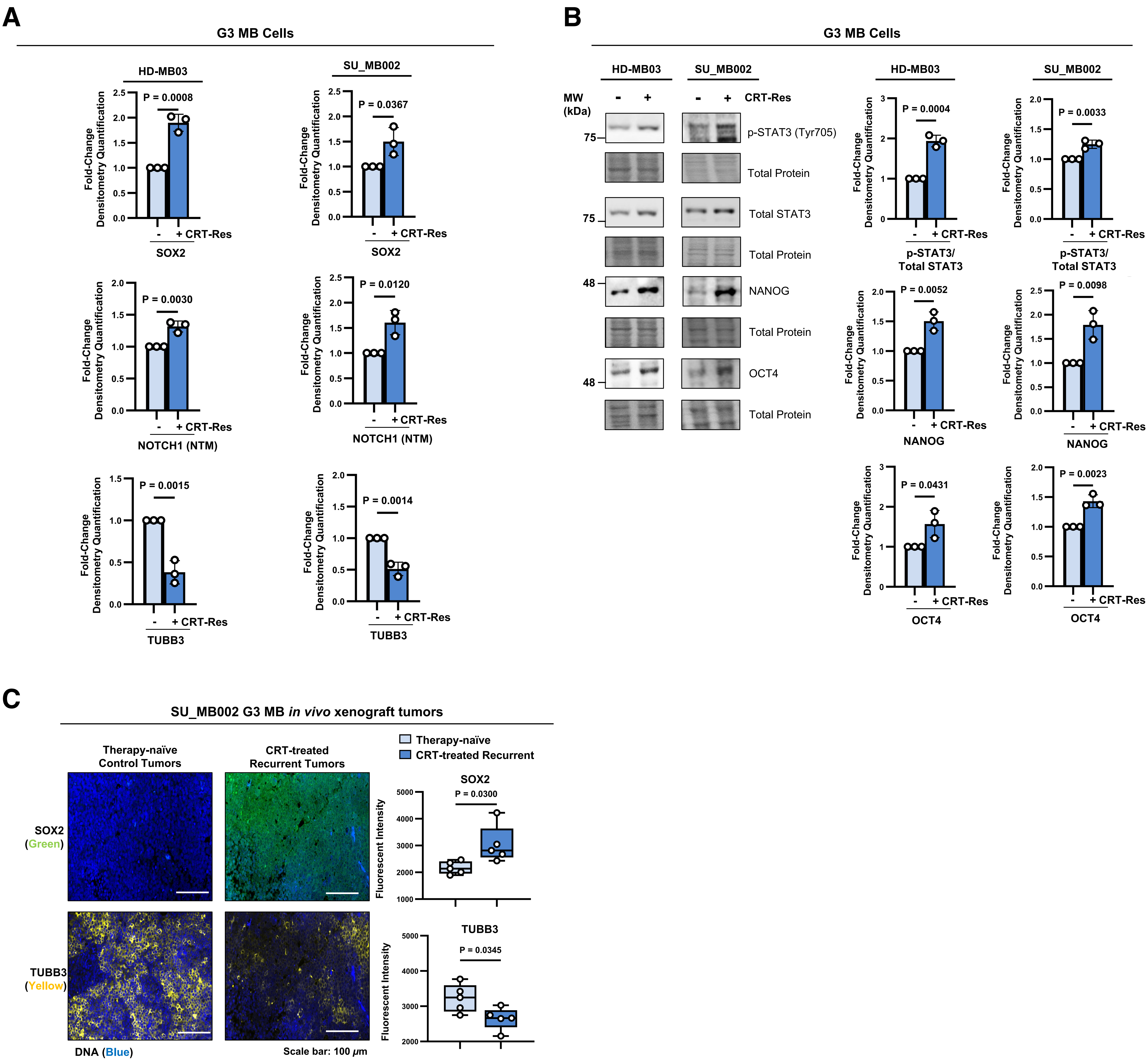


**Supplementary Figure 3**. **(A)** Graphs represent densitometry quantification of the blots presented in Figure 4A, measurement normalized to the total protein of SOX2, NOTCH1 (NTM), and TUBB3 in therapy naïve and CRT-Res HD-MB03 and SU_MB002 cells from *n* = 3 experimental replicates, presented as mean + s.e.m; unpaired two-tailed t test. **(B)** Immunoblot of p-Stat3, total Stat3, Nanog, and OCT3/4 expression in CRT-Res HD-MB03 and SU_MB002 cells. Graphs represent densitometry quantification measurement normalized to the total protein of p-Stat3/total Stat3, Nanog, and OCT3/4 in therapy naïve and CRT-Res HD-MB03 and SU_MB002 cells from *n* = 3 experimental replicates, presented as mean + s.e.m; unpaired two-tailed t test. **(C)** Representative IF images of SU_MB002 xenograft tumors from therapy-naïve controls and CRT-treated recurrent groups ed for SOX2 (green), TUBB3 (yellow), and DNA  DAPI (blue). Graphs represent quantification of fluorescent intensity from n = 5 tumor samples per group, with a solid line at the mean; unpaired two-tailed t test

# Supplementary Figure 4

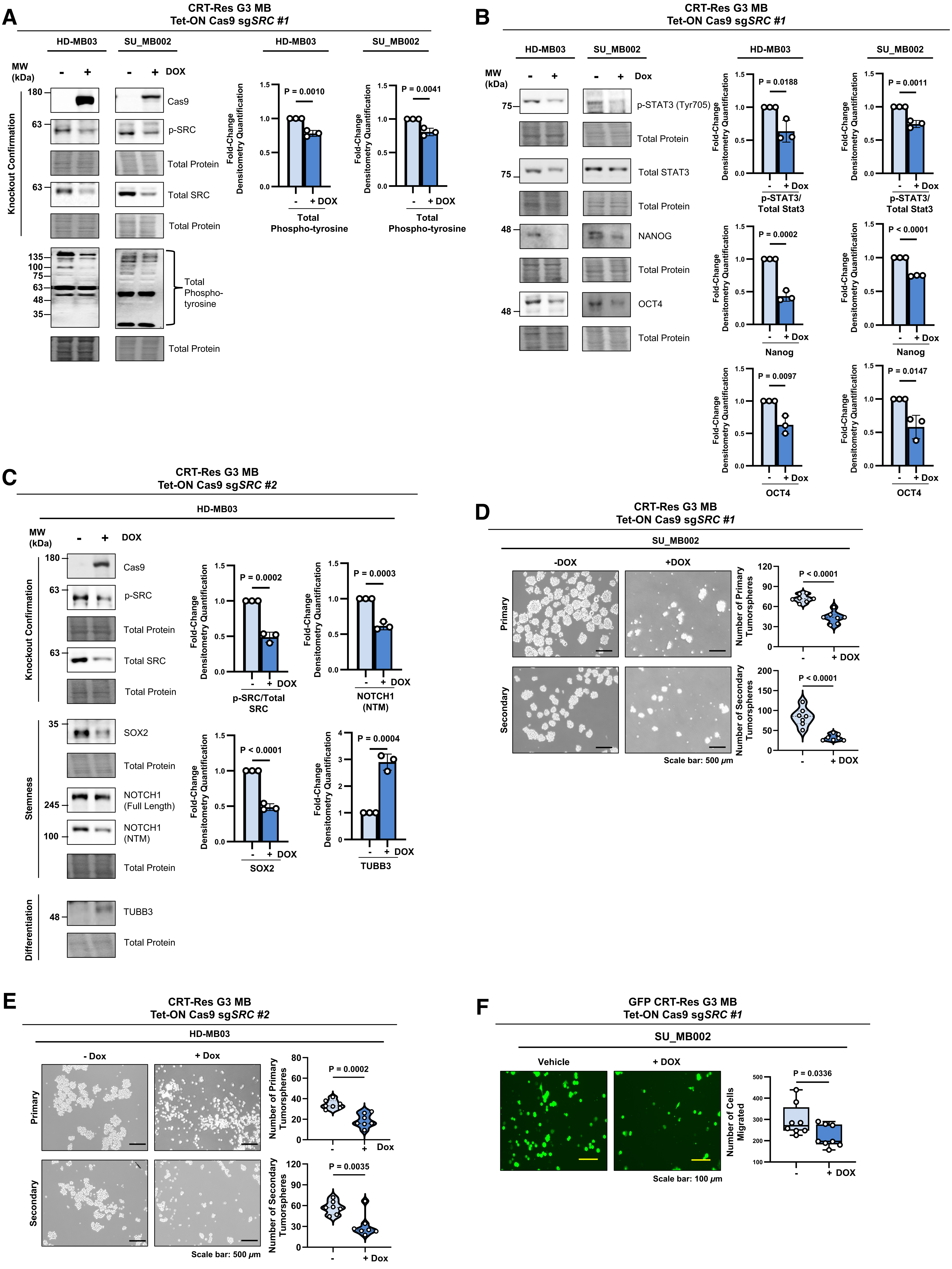

**Supplementary Figure 4**. **(A)** Immunoblot of Cas9, p-SRC (Y419), total SRC and Anti-Phospho tyrosine in CRT-Res HD-MB03 and SU_MB002 cells expressing Tet-ON Cas9 sg*SRC* #1 treated with DOX. Graphs represent densitometry quantification measurement of Anti-Phospho Tyrosine normalized to the total protein levels from *n* = 3 experimental replicates, presented as mean ± s.e.m; unpaired two-tailed t test. **(B)** Immunoblot of p-Stat3, total Stat3, Nanog, and OCT3/4 expression in CRT-Res HD-MB03 and SU_MB002 cells expressing Tet-ON Cas9 sg*SRC* #1 treated with DOX. Graphs represent densitometry quantification measurement normalized to the total protein of p-Stat3/total Stat3, Nanog, and OCT3/4 in therapy naïve and CRT-Res HD-MB03 and SU_MB002 cells from *n* = 3 experimental replicates, presented as mean + s.e.m; unpaired two-tailed t test**.(C)** Immunoblots of Cas9, p-SRC (Y419), total SRC, SOX2, NOTCH1 (full length and NTM), and TUBB3 in CRT-Res HD-MB03 cells expressing Tet-ON Cas9 sg*SRC* #2 treated with DOX to induce *SRC* KO. Graphs represent densitometry quantification measurement of p-SRC/Total SRC, SOX2, NOTCH1 (NTM), and TUBB3 normalized to the total protein levels from *n* = 3 experimental replicates, presented as mean ± s.e.m; unpaired two-tailed t test. **(D)** Primary and secondary tumorsphere formation assay from CRT-Res SU_MB002 cells expressing Tet-ON Cas9 sg*SRC* #1 treated with DOX to induce *SRC* KO. Violin plot represents quantification of total sphere number from *n* =7 replicates, with dashed lines at the mean and quartiles; unpaired two-tailed t test. **(E)** Primary and secondary tumorsphere formation assay from CRT-Res HD-MB03 cells expressing Tet-ON Cas9 sg*SRC* #2 treated with DOX to induce *SRC* KO. Violin plot represents quantification of total sphere number from n =7 replicates, with dashed lines at the mean and quartiles; unpaired two-tailed t test. **(F)** Representative images and quantification of migration assays in SU_MB002 cells expressing Tet-ON Cas9, sg*SRC* #1, and GFP, treated with DOX to induce *SRC* KO. Box-and-whisker plots represent quantification of migrated cells from *n* = 8 replicates, with a solid line at the mean, unpaired two-tailed t test.

# Supplementary Figure 5

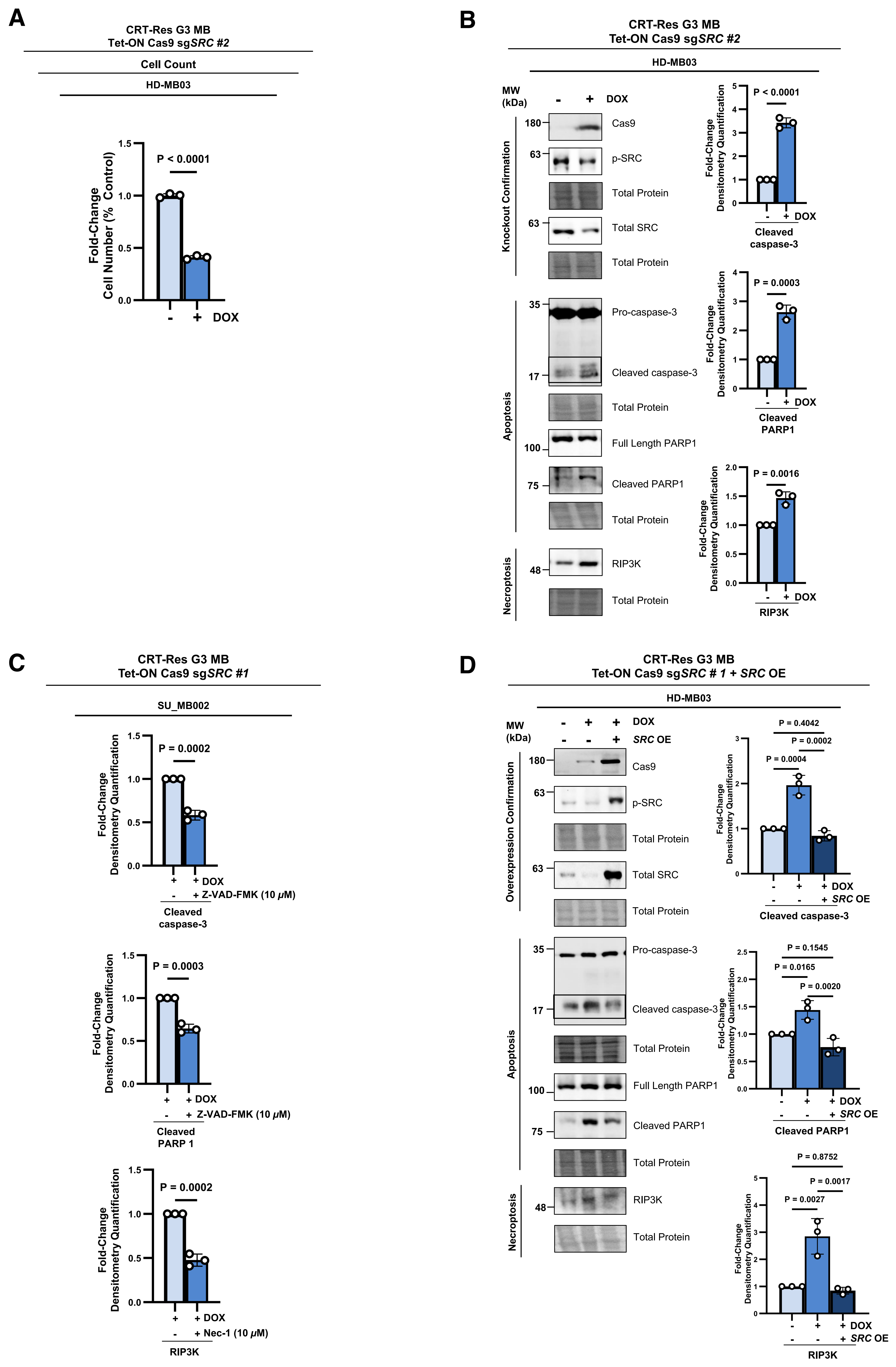

**Supplementary Figure 5. (A)** Cell count analysis of CRT-Res HD-MB03 cells expressing Tet-ON Cas9 sg*SRC #2* treated with DOX to induce *SRC* KO. Graph represents fold-change in cell number of *SRC* KO cells (+DOX) compared to controls (-DOX). Data presented as mean ± s.e.m; from *n* = 3 experimental replicates; unpaired two-tailed t test**. (B)** Immunoblot of Cas9, p-SRC (Y419), total SRC, pro-caspase-3, cleaved caspase-3, full length PARP1, cleaved PARP1, and RIP3K in CRT-Res HD-MB03 cells expressing Tet-ON Cas9 sg*SRC #2* treated with DOX. Graphs represent densitometry quantification measurement of cleaved caspase-3, cleaved PARP1, and RIP3K normalized to the total protein levels from *n* = 3 experimental replicates, presented as mean ± s.e.m; unpaired two-tailed t test. **(C)** Graphs represent densitometry quantification of blots shown in Figure 5C, measurement of cleaved caspase-3, cleaved PARP1, and RIP3K normalized to the total protein levels in CRT-Res SU_MB002 cells expressing Tet-ON Cas9 sgSRC #1 following DOX-induced SRC KO treated with Z-VAD-FMK (10 µM) or Nec-1 (10 µM) treatment from n = 3 experimental replicates, presented as mean ± s.e.m; unpaired two-tailed t test. **(D)** Immunoblot of Cas9, p-SRC (Y419), total SRC, pro-caspase-3, cleaved caspase-3, full length PARP1, cleaved PARP1, and RIP3K in CRT-Res HD-MB03 cells expressing Tet-ON Cas9 sg*SRC #1* treated with DOX and Transfected with *SRC* OE. Graphs represent densitometry quantification measurement of cleaved caspase-3, cleaved PARP1, and RIP3K normalized to the total protein levels from *n* = 3 experimental replicates, presented as mean ± s.e.m; unpaired two-tailed t test.

# Supplementary Figure 6

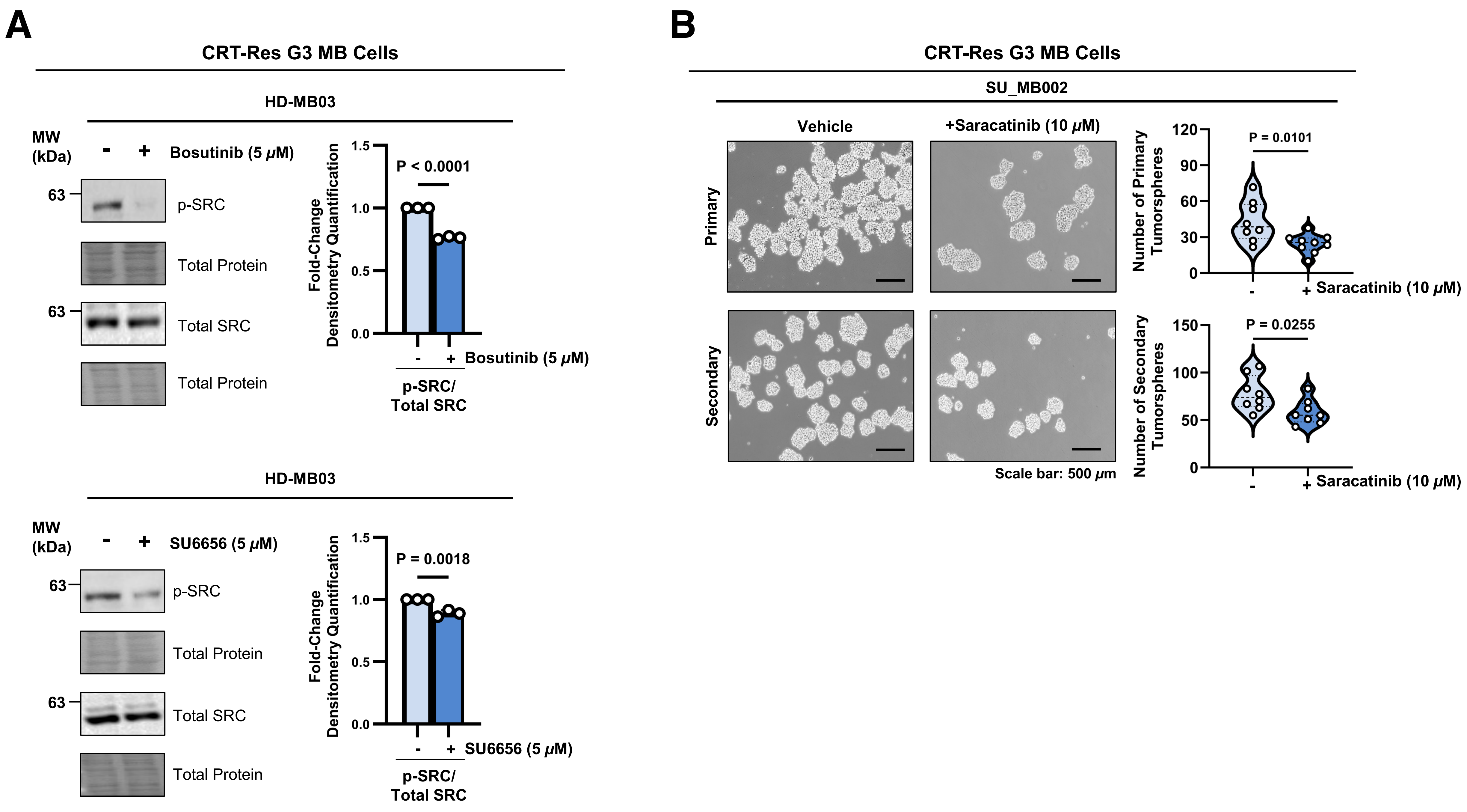


**Supplementary Figure 6. (A)** Immunoblot of p-SRC (Y419) and total SRC in CRT-Res HD-MB03 cells treated with SRC inhibitors: Bosutinib (top panel) and SU6656 (bottom panel). Graphs represent densitometry quantification measurement of p-SRC/total SRC normalized to the total protein from $n$ = 3 experiments, presented as mean ± s.e.m.; unpaired two-tailed t tests. **(B)** Primary and secondary tumorsphere formation assay of CRT-Res SU_MB002 cells treated with Saracatinib. Violin plot represents quantification of total sphere number from $n$ = 8 experiments, with dashed lines at the mean and quartiles; unpaired two-tailed t test.

# Supplementary Figure 7

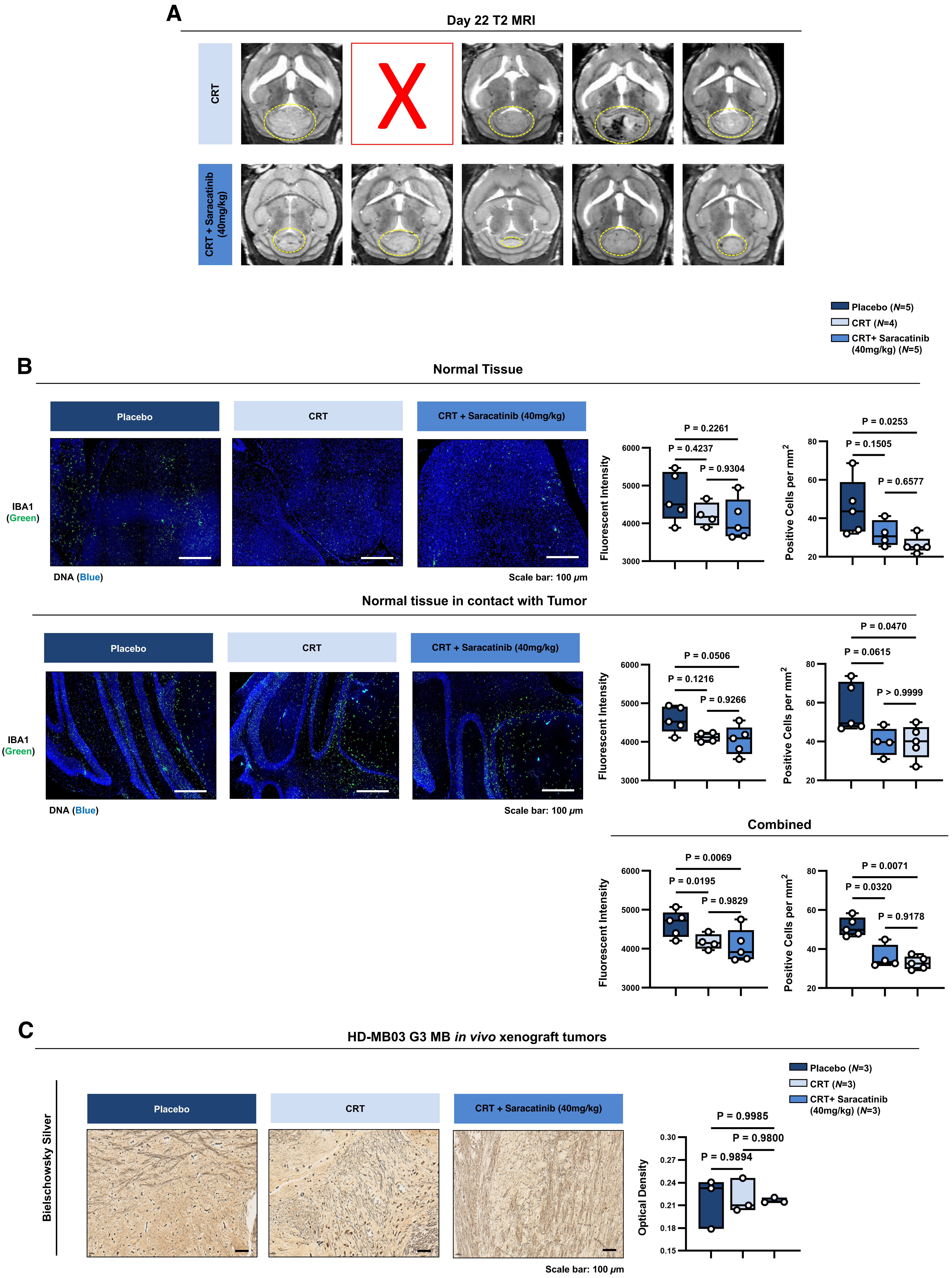

**Supplementary Figure 7**. **(A)** Day 22 post-engraftment T2-weighted MRI scans show brain tumors (yellow dashed outlines) in representative mice treated with either CRT alone or CRT + Saracatinib. Red "X" indicates mice removed from the study due to complications unrelated to the experimental treatment conditions. **(B)** Representative IF images of IBA1 (Green) and DAPI-ed DNA (blue) in HD-MB03 therapy-naïve control tumors, CRT-treated, and CRT + Saracatinib-treated recurrent tumors. Box-and-whisker plots represent quantification of fluorescence intensity from $n$ = 5 (therapy-naïve), $n$ = 4 (CRT alone) and $n$ = 5 (CRT + Saracatinib) animals per group, with solid lines at the mean; mean + s.e.m. two-way ANOVA with Tukey's test. **(C)** Representative Bielschowsky Silver images in HD-MB03 therapy-naïve control tumors, CRT-treated, and CRT + Saracatinib-treated recurrent tumors. Graph represents quantification of optical density from $n$ = 3 tumor samples per group, with a solid line at the mean, mean + s.e.m. two-way ANOVA with Tukey's test.
