# Supplementary material for: Targeting SRC enhances differentiation and promotes multifaceted cell death mechanisms in recurrent group 3 medulloblastoma
Source: Cell Death Dis. 2026 Apr 24;17(1):543. doi: 10.1038/s41419-026-08751-9 (PMC13237022; doi:10.1038/s41419-026-08751-9)

SUPPLEMENTARY FILE - UNCROPPED ORIGINAL BLOTS

Targeting SRC enhances differentiation and promotes multifaceted cell death mechanisms in recurrent group 3 medulloblastoma

Kuzmychova H., Chawla U. & Martell E. *et al*

Figure 1

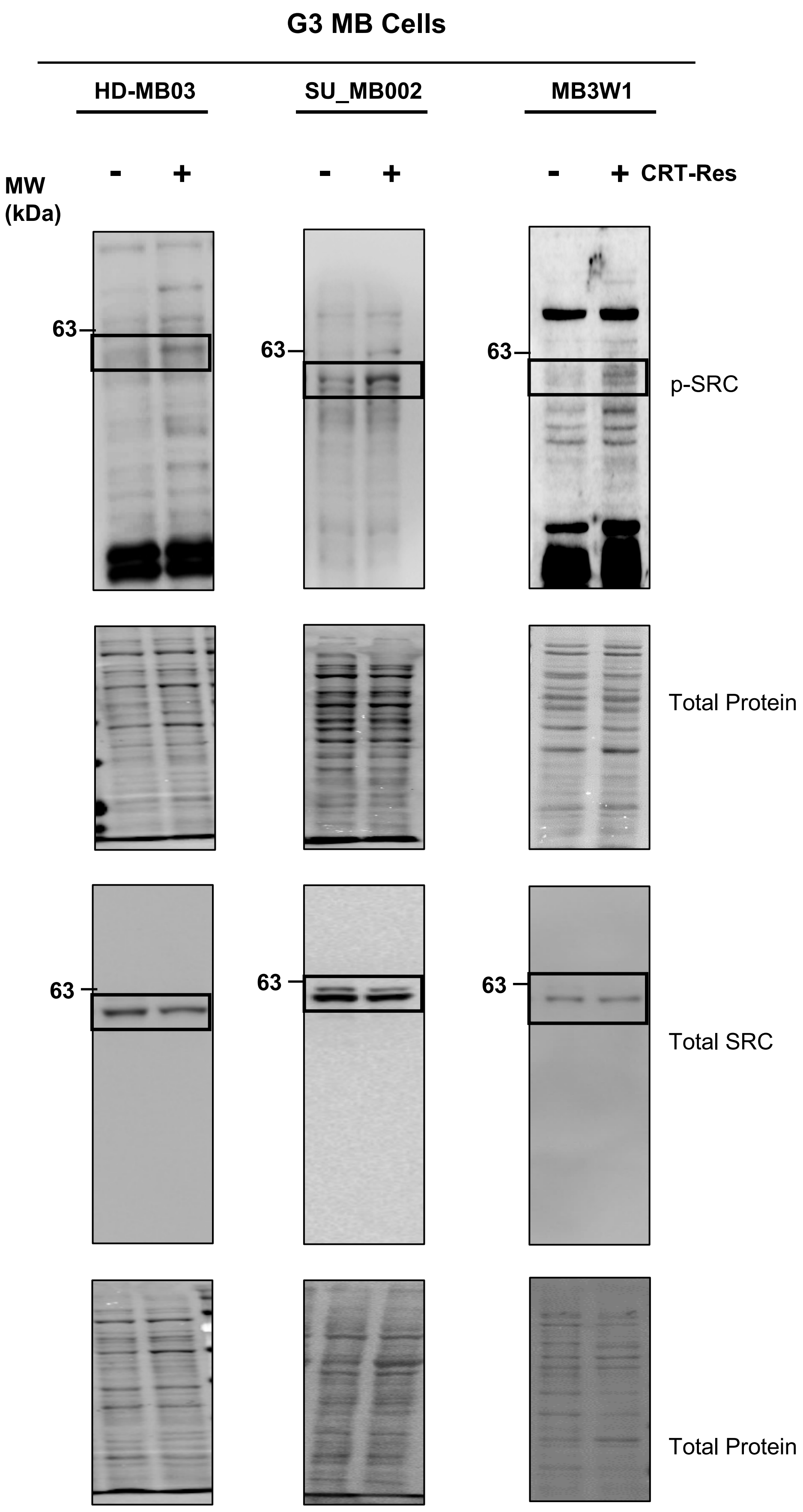
G3 MB Cells
HD-MB03
SU_MB002
MB3W1
MW
(kDa)
- + - + - + CRT-Res
63
p-SRC
Total Protein
63
Total SRC
Total Protein

Figure 2

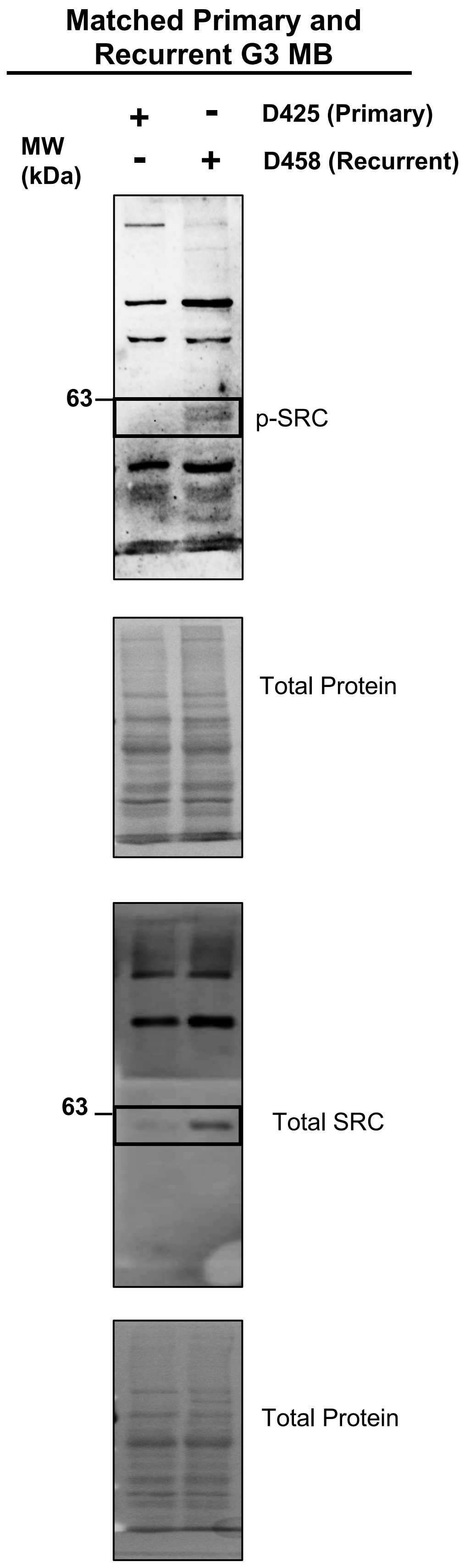

Matched Primary and
Recurrent G3 MB
+ -
D425 (Primary)
MW
(kDa)
- +
D458 (Recurrent)
63
p-SRC
Total Protein
63
Total SRC
Total Protein

Figure 3

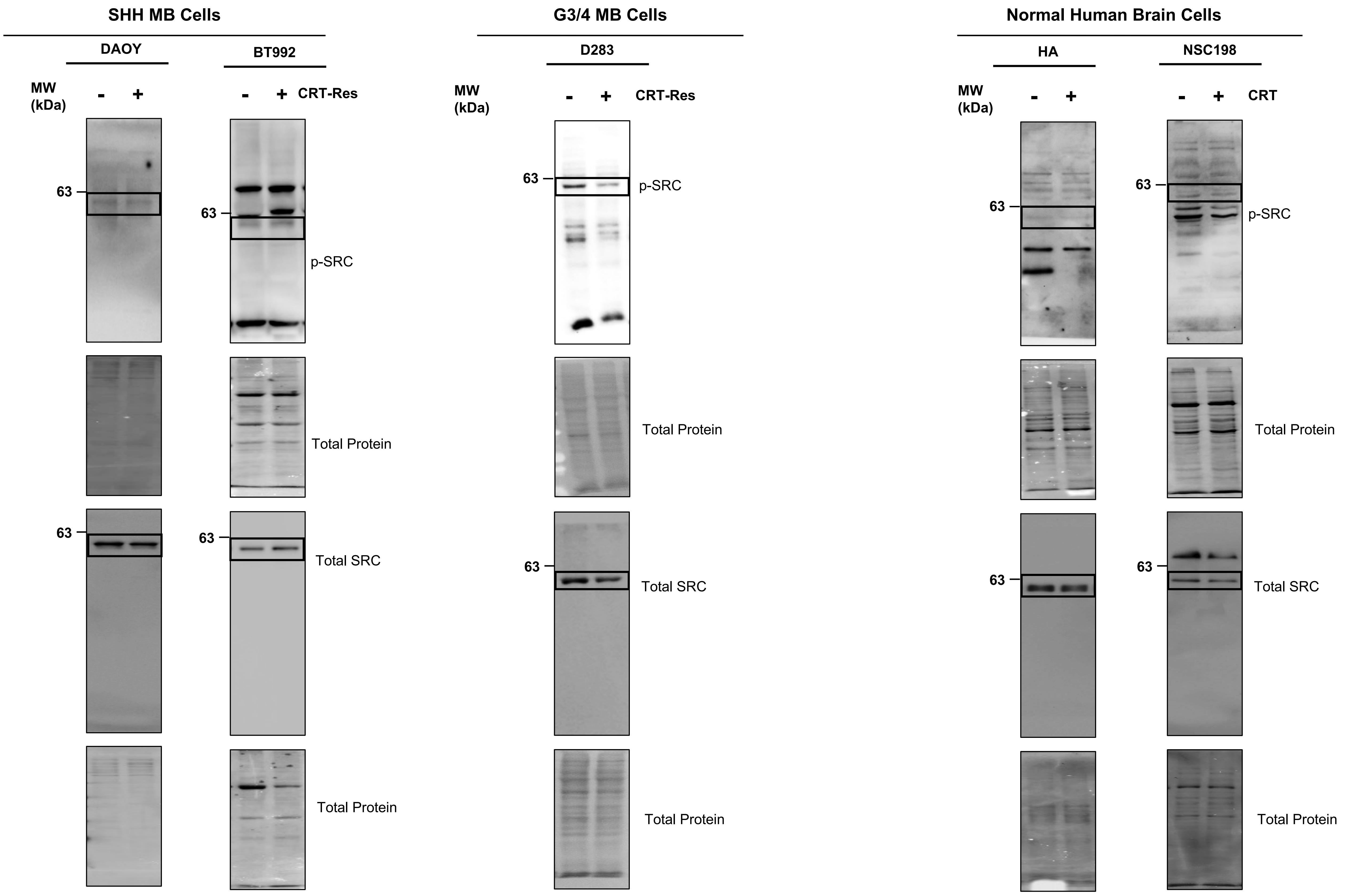
SHH MB Cells
DAOY
BT992
G3/4 MB Cells
D283
Normal Human Brain Cells
HA
NSC198
MW (kDa)
- +
CRT-Res
CRT
63
p-SRC
Total Protein
Total SRC
Total Protein

Figure 4

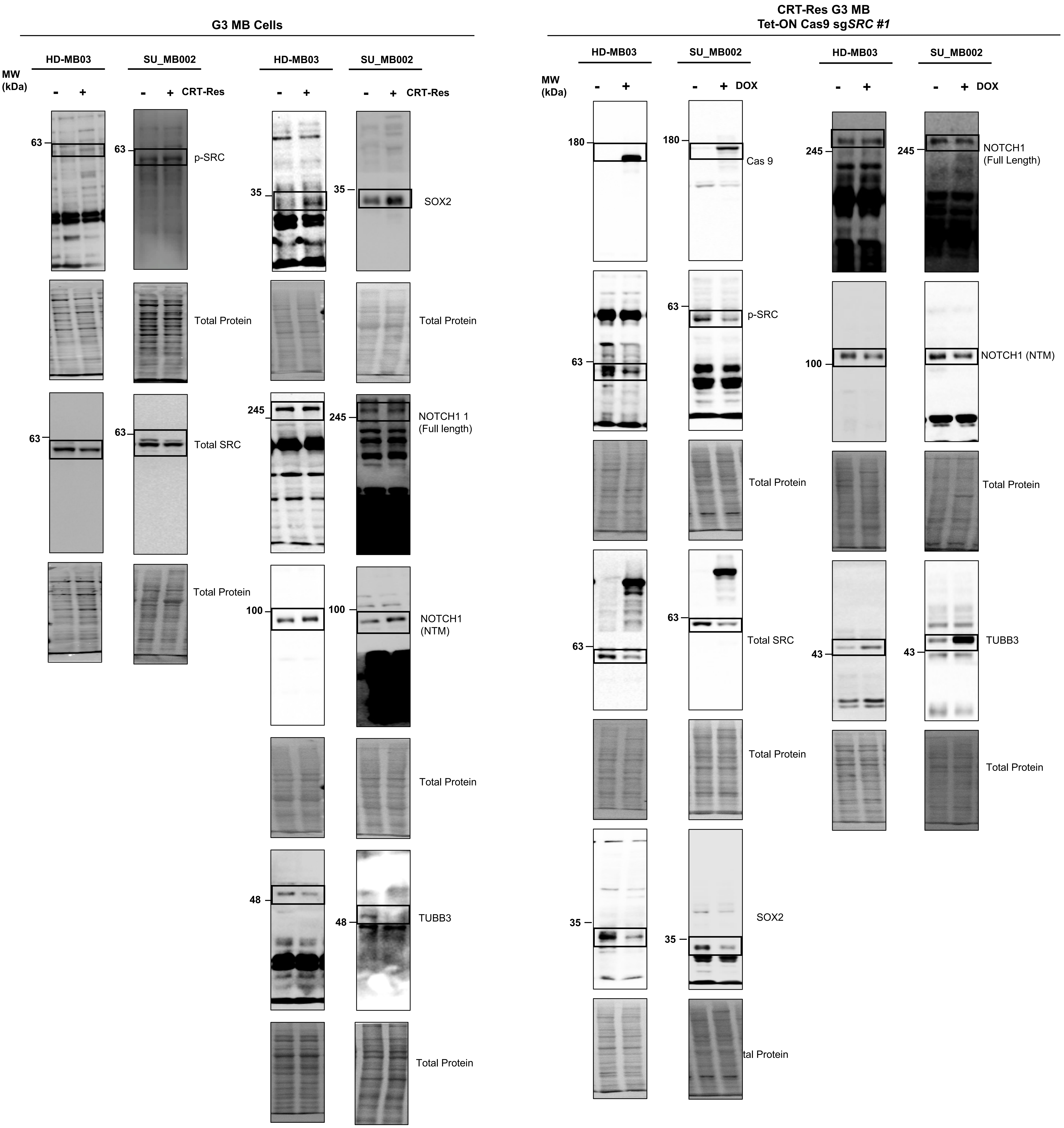
G3 MB Cells
CRT-Res G3 MB
Tet-ON Cas9 sg*SRC #1*
HD-MB03
SU_MB002
MW (kDa)
- + CRT-Res
- + DOX
p-SRC
SOX2
Total Protein
Total SRC
NOTCH1 1 (Full length)
NOTCH1 (NTM)
TUBB3
Cas 9
NOTCH1 (Full Length)
63
35
245
100
48
180
43
tal Protein

Figure 4

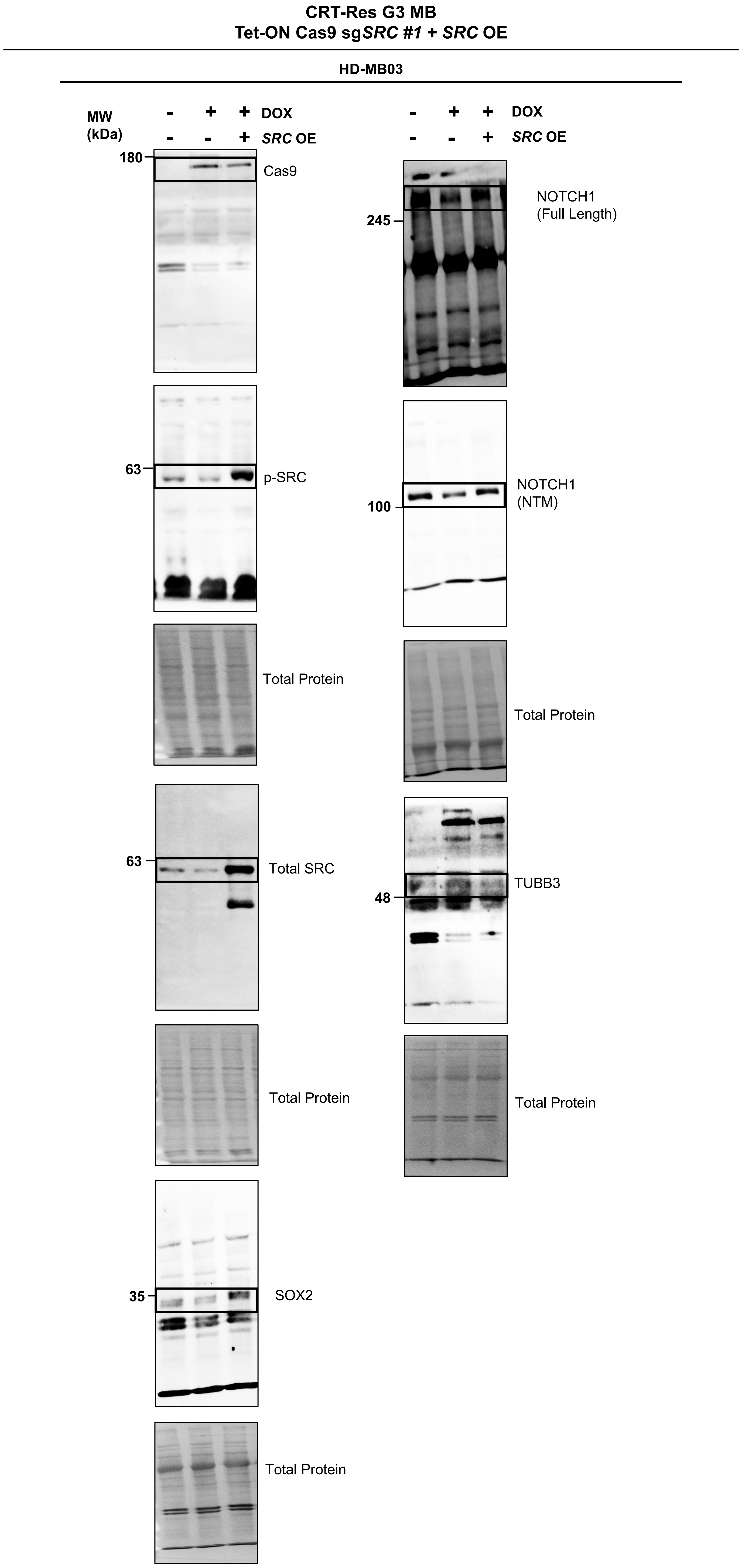
CRT-Res G3 MB
Tet-ON Cas9 sg*SRC #1* + *SRC* OE
HD-MB03
MW (kDa)
DOX
SRC OE
180
Cas9
63
p-SRC
Total Protein
63
Total SRC
Total Protein
35
SOX2
Total Protein
245
NOTCH1 (Full Length)
100
NOTCH1 (NTM)
Total Protein
48
TUBB3
Total Protein

Figure 5

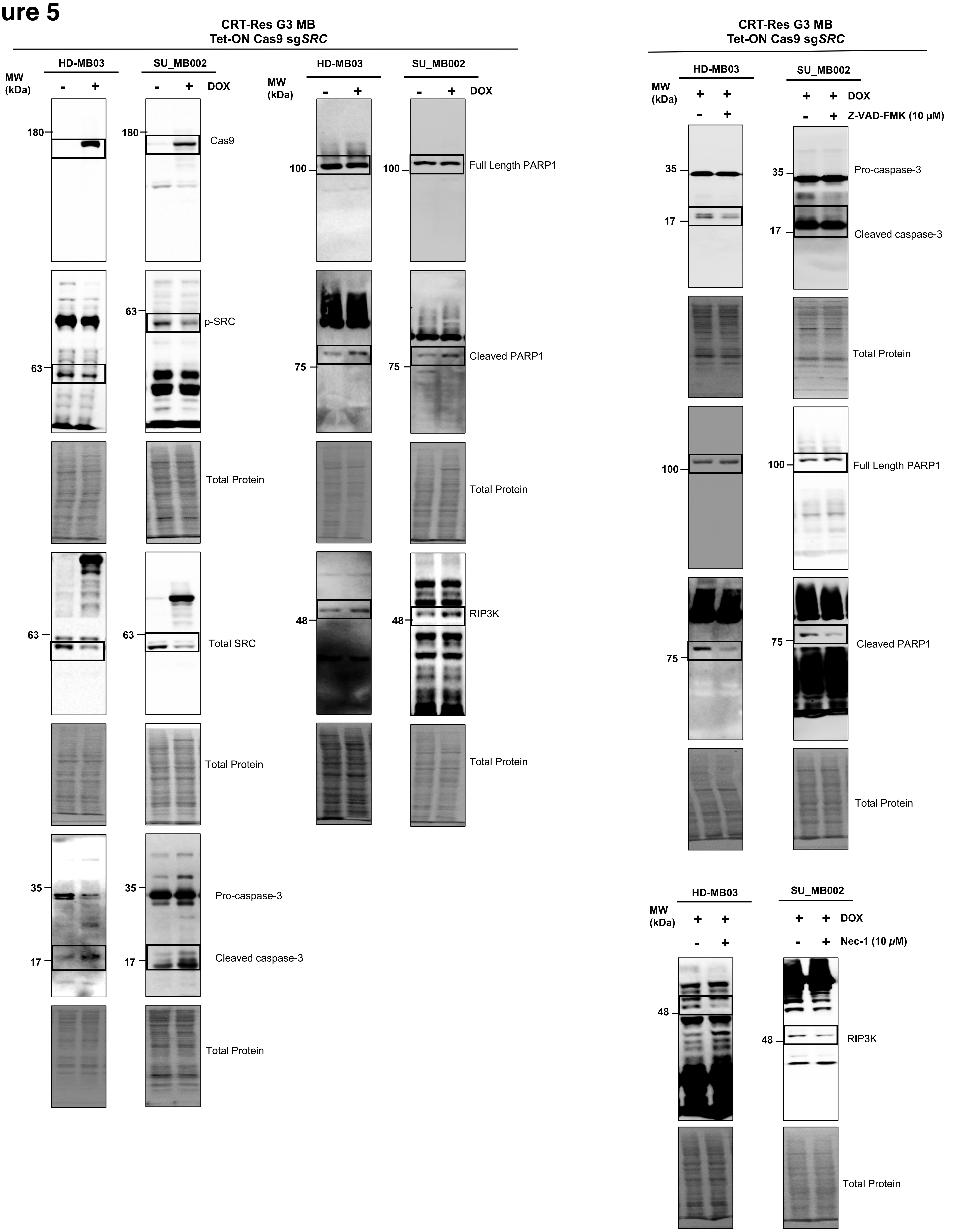

CRT-Res G3 MB
Tet-ON Cas9 sgSRC
HD-MB03
SU_MB002
MW (kDa)
DOX
Z-VAD-FMK (10 μM)
Nec-1 (10 μM)
Cas9
p-SRC
Total Protein
Total SRC
Pro-caspase-3
Cleaved caspase-3
Full Length PARP1
Cleaved PARP1
RIP3K
180
100
75
63
48
35
17

Figure 6

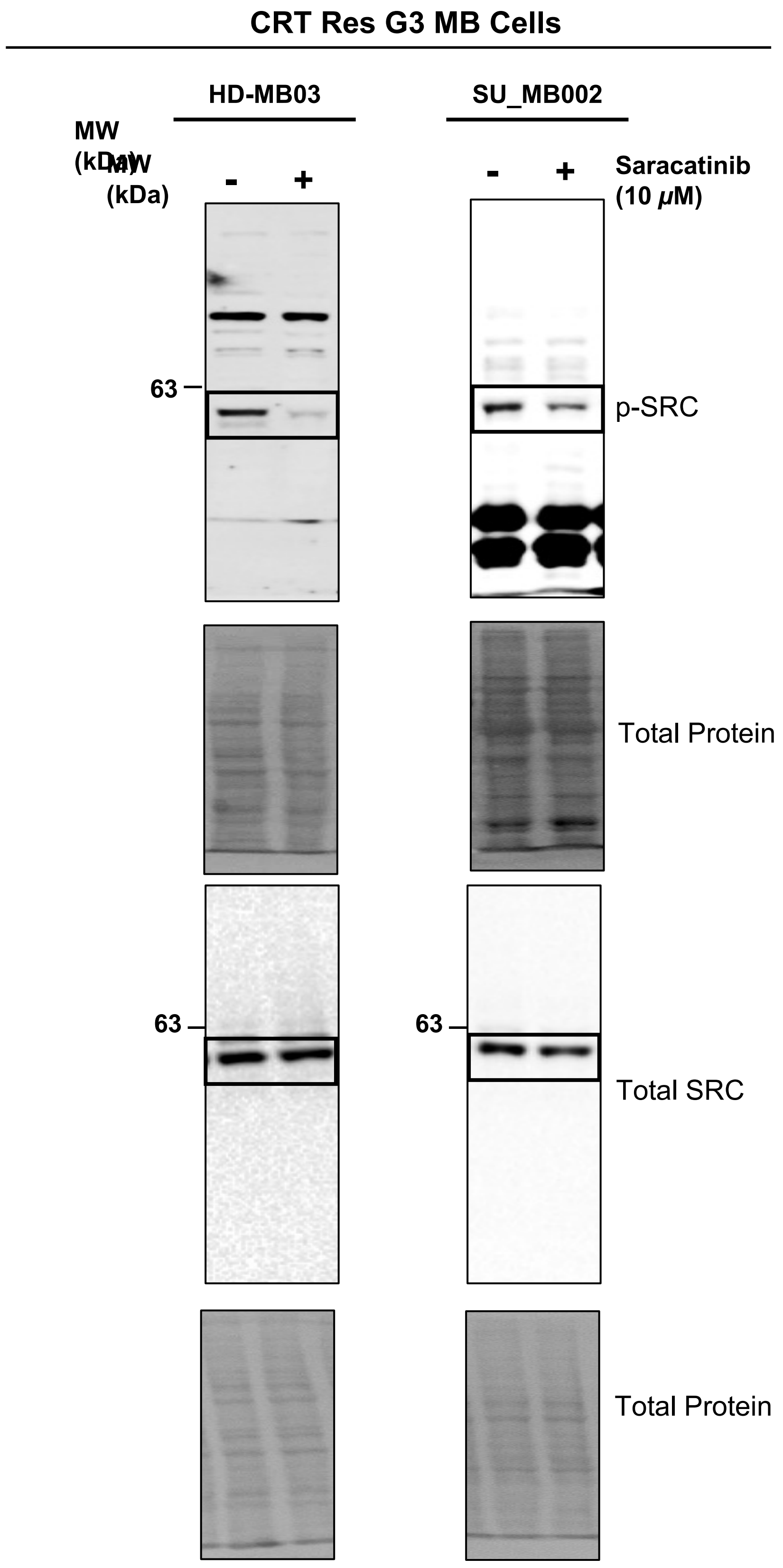
CRT Res G3 MB Cells
HD-MB03
SU_MB002
MW (kDa)
MW (kDa)
- +
- +
Saracatinib (10 μM)
63
p-SRC
Total Protein
63
63
Total SRC
Total Protein

Figure 7

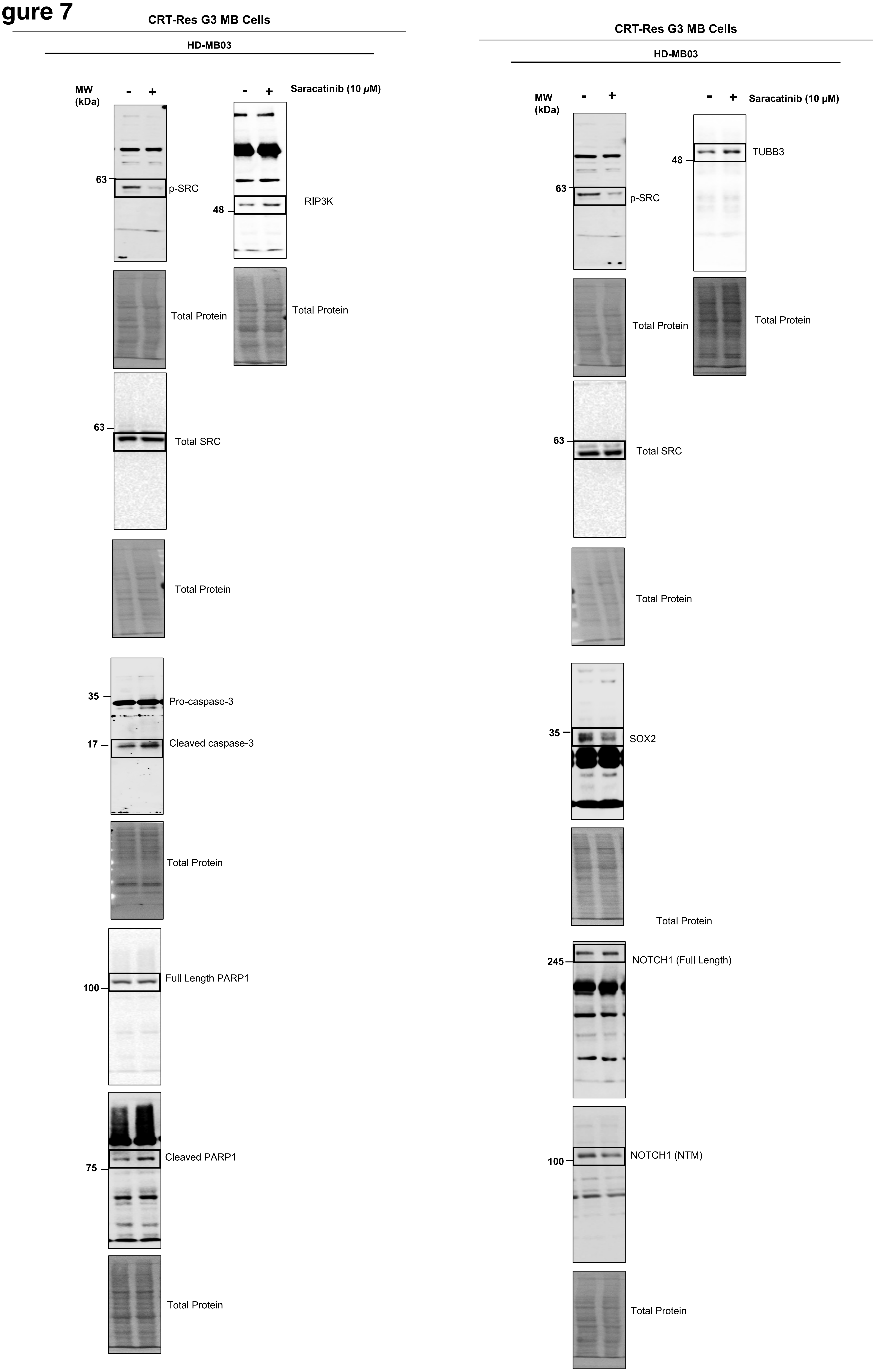

CRT-Res G3 MB Cells
HD-MB03
MW (kDa)
- +
- +
Saracatinib (10 μM)
63
p-SRC
48
RIP3K
Total Protein
Total Protein
63
Total SRC
Total Protein
35
Pro-caspase-3
17
Cleaved caspase-3
Total Protein
Full Length PARP1
100
Cleaved PARP1
75
Total Protein
CRT-Res G3 MB Cells
HD-MB03
MW (kDa)
- +
- +
Saracatinib (10 μM)
48
TUBB3
63
p-SRC
Total Protein
Total Protein
63
Total SRC
Total Protein
35
SOX2
Total Protein
245
NOTCH1 (Full Length)
100
NOTCH1 (NTM)
Total Protein

Supplementary Figure 2

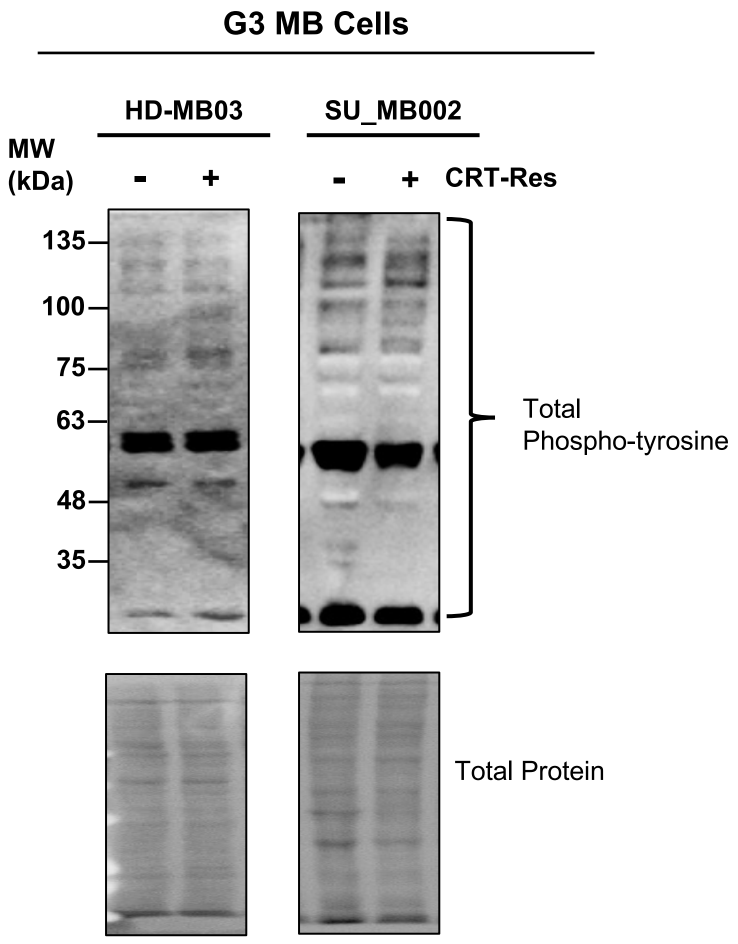

Supplementary Figure 3

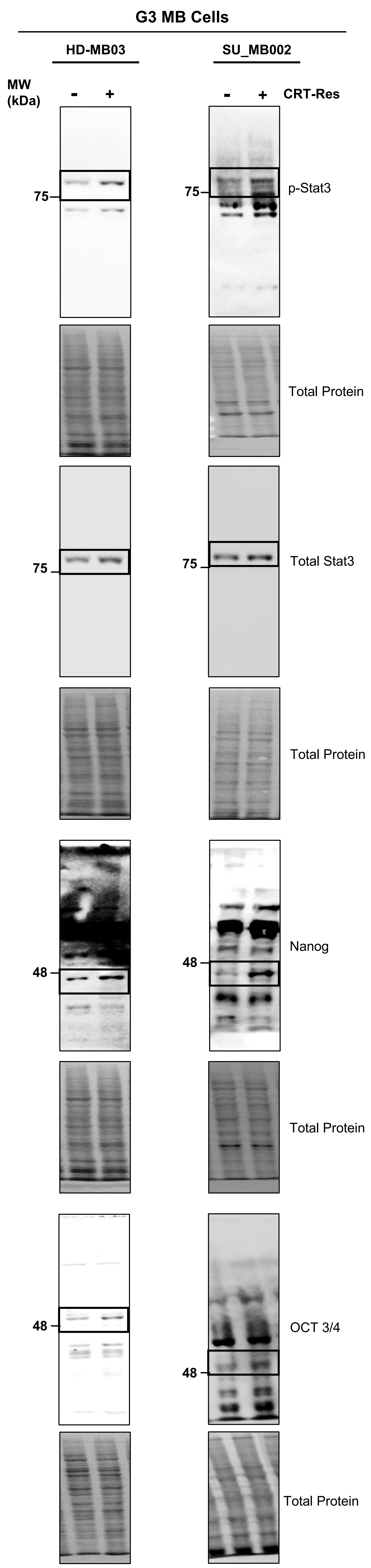

Supplementary Figure 4

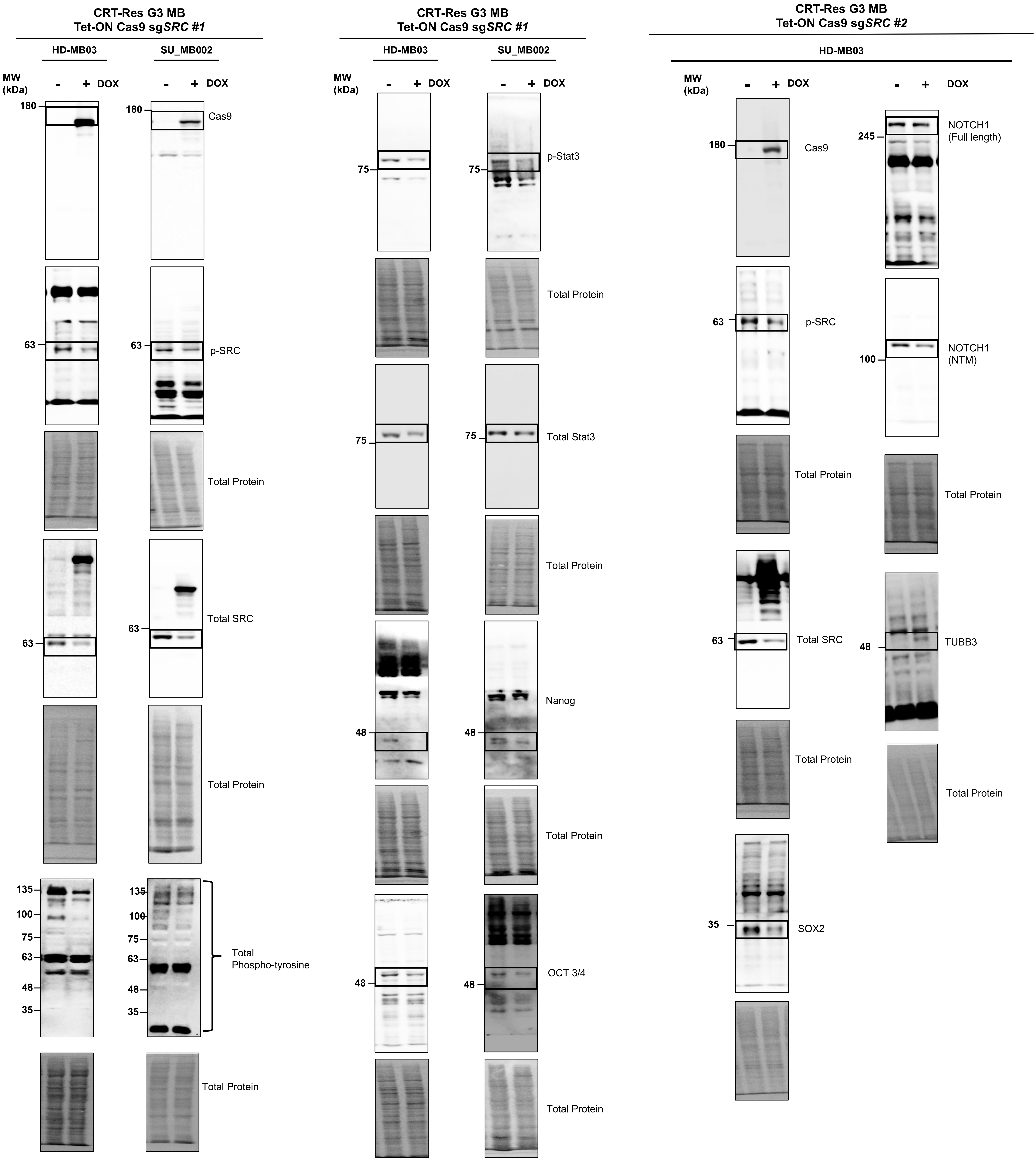

Supplementary Figure 5

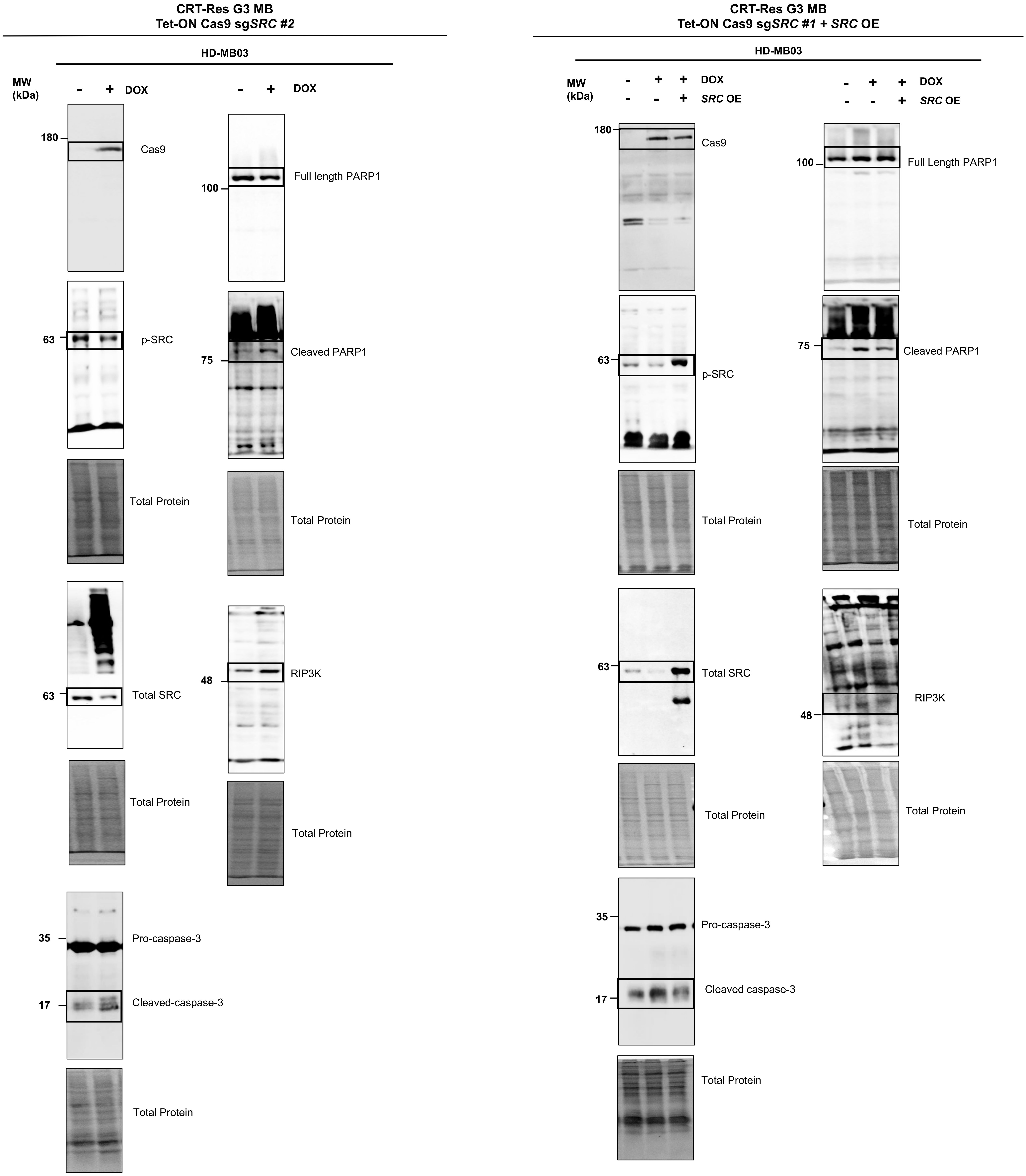

Supplementary Figure 6

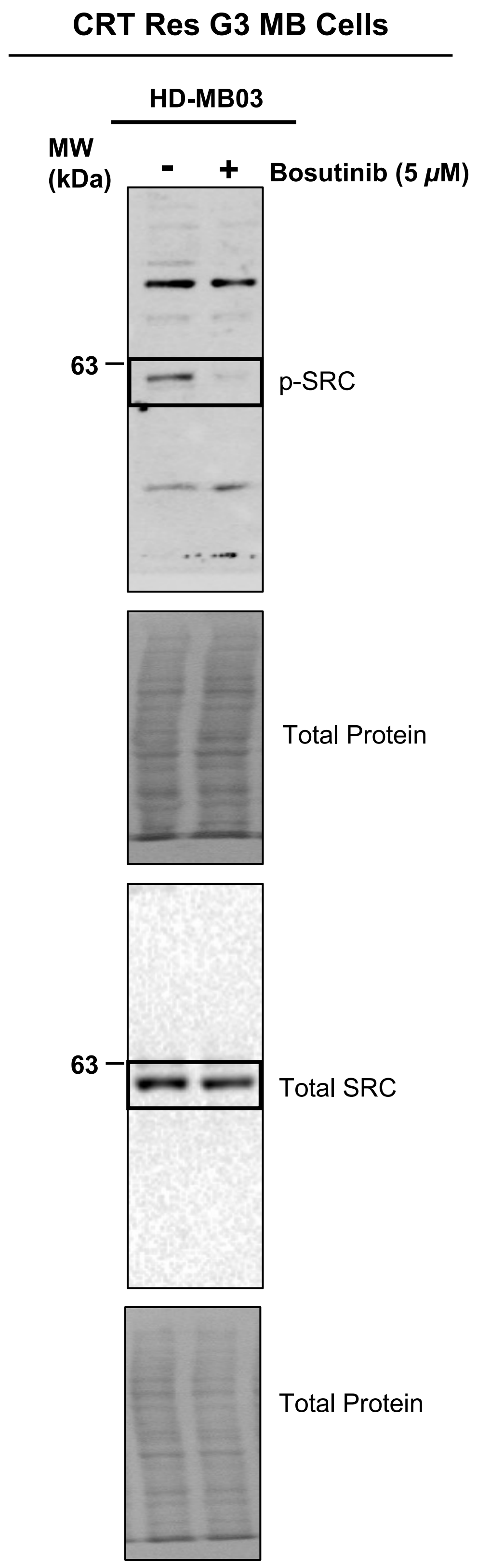

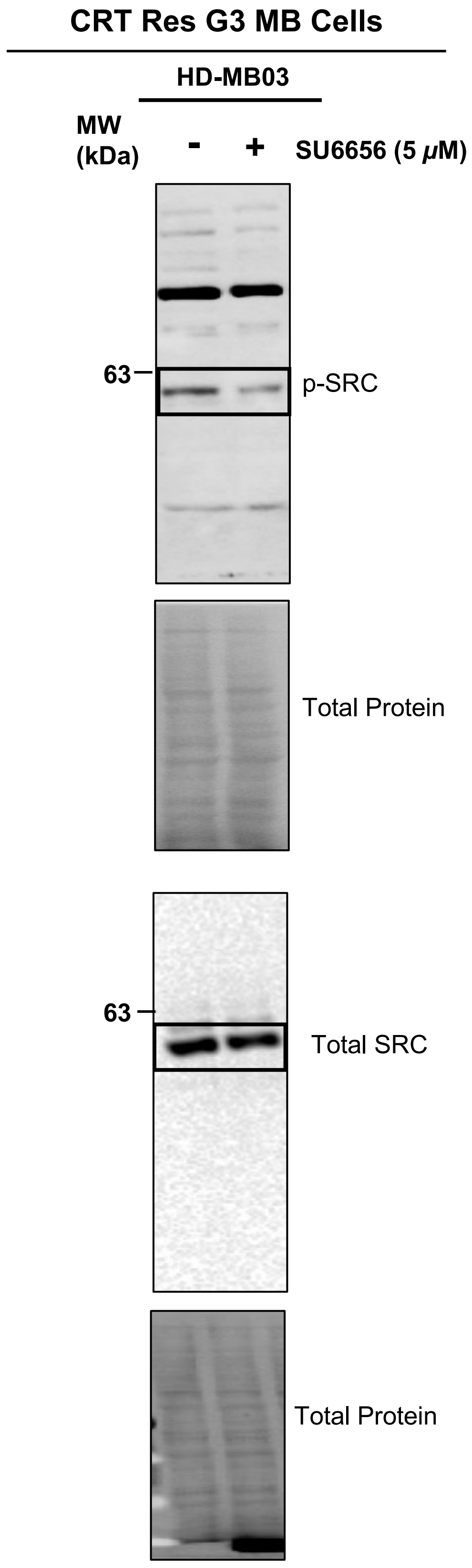

Supplement: Supplementary file 4 — Uncropped Western Blots [file 41419_2026_8751_MOESM4_ESM.pdf]
